# Roles of Embryonic Lethal Abnormal Vision-Like RNA Binding Proteins in Cancer and Beyond

**DOI:** 10.3389/fcell.2022.847761

**Published:** 2022-04-06

**Authors:** Haijian Cai, Dandan Zheng, Yizhu Yao, Lehe Yang, Xiaoying Huang, Liangxing Wang

**Affiliations:** The First Affiliated Hospital, Wenzhou Medical University, Wenzhou, China

**Keywords:** ELAVL proteins, RNA binding proteins, non-coding RNAs, post-transcriptional regulation, cancers

## Abstract

Embryonic lethal abnormal vision-like (ELAVL) proteins are RNA binding proteins that were originally discovered as indispensable regulators of the development and functioning of the nervous system. Subsequent studies have shown that ELAVL proteins not only exist in the nervous system, but also have regulatory effects in other tissues. ELAVL proteins have attracted attention as potential therapeutic targets because they stabilize multiple mRNAs by binding within the 3′-untranslated region and thus promote the development of tumors, including hepatocellular carcinoma, pancreatic cancer, ovarian cancer, breast cancer, colorectal carcinoma and lung cancer. Previous studies have focused on these important relationships with downstream mRNAs, but emerging studies suggest that ELAVL proteins also interact with non-coding RNAs. In this review, we will summarize the relationship of the ELAVL protein family with mRNA and non-coding RNA and the roles of ELAVL protein family members in a variety of physiological and pathological processes.

## Introduction

**FIGURE 1:**
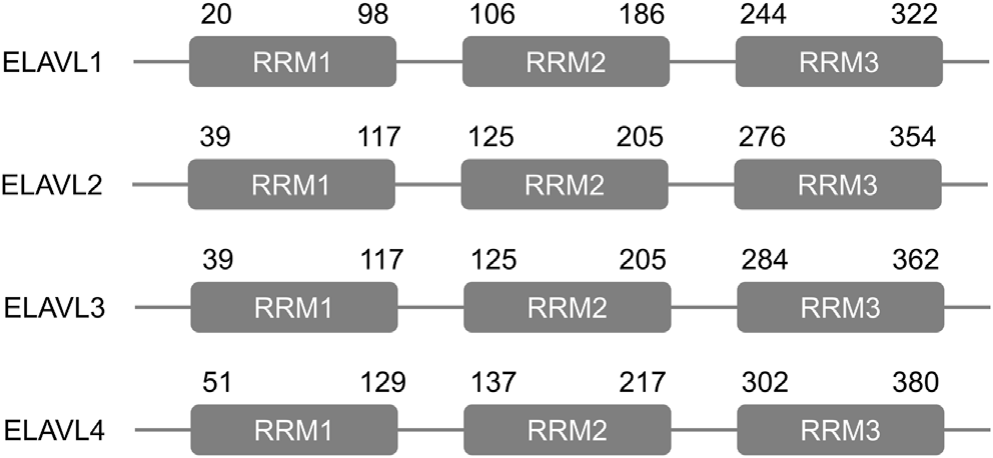
Structure of the human ELAVL proteins. Each ELAVL protein consists of three similar and conserved RNA recognition motifs (RRMs). The numbers indicate amino acid positions.

The embryonic lethal abnormal vision-like (ELAVL) proteins in fish, frogs, and mammals are defined as RNA-binding proteins (RBPs), and they play important roles in post-transcriptional regulation (Campos et al., 1985). ELAVL proteins were first discovered in *Drosophila* due to their ability to interact with AU-rich element (ARE)-containing transcripts (Campos et al., 1985). Each member of the ELAVL protein family, which includes ELAVL1-4 (HuR HuB, HuC, HuD) (Figure 1), consists of three similar and conserved RNA recognition motifs (RRM) (Toba and White, 2008; Colombrita et al., 2013). The sequence of a hinge region between RRMs 2 and 3 differs among the four family members (Good, 1995), and its presence is key to the ability of these proteins to shuttle into and out of the nucleus (Fan and Steitz, 1998a).

Biologically, ELAVL proteins were originally discovered as indispensable regulators of nervous system development and physiological function (Rogulja-Ortmann et al., 2014; Wang et al., 2019a; Zhao et al., 2020a). Interestingly, subsequent studies have shown that they not only exist in the nervous system, but also have regulatory effects in other tissues, including hepatocytes (Wang et al., 2021), fat cells (Siang et al., 2020), vascular smooth muscle cells (Liu et al., 2020a), and intestinal epithelial cells (Liu et al., 2019).

Previous studies have mainly focused on the relationship between ELAVL proteins and downstream mRNA transcripts, and less attention has been paid to interactions between ELAVLs and non-coding RNA (ncRNA) molecules. The regulation and metabolism of ncRNA is an emerging research topic, and ncRNAs have been shown to play important roles in a variety of fields, such as cancer (Deschenes-Furry et al., 2007; Schultz et al., 2020), inflammation (Wen et al., 2021), and cell differentiation (Chen et al., 2021a). This review aims to summarize relationships of the ELAVL family of proteins with mRNAs and ncRNAs and the regulation of ELAVL family proteins in various physiological and pathological processes.

## Members of the ELAVL Family

ELAVL1, also known as human antigen R (HuR), has been established as a tumor-specific antigen in colorectal carcinoma (Wang et al., 2000) and tumors of the central nervous system (Nabors et al., 2001). This protein is a widely expressed RBP whose function in many cell types has been elucidated. The gene encoding human ELAVL1 is localized to chromosome 19p13.2, and while the protein is mainly localized to the nucleus, it will translocate to the cytoplasm following stimulation by a variety of extracellular stimuli (Chand et al., 2017).

The consequences for an mRNA after ELAVL1 binding to its ARE depend on the mRNA itself and the cell type. When the target ARE, which usually contains multiple AUUUA repeats, appears in the 3′-untranslated regions (UTRs) of an mRNA, ELAVL1 binding often results in mRNA instability (Fan et al., 1997), and thus selective binding of ELAVL1 to the ARE at the 3′ end of an mRNA can lead to rapid degradation (Myer et al., 1997). However, in most cases, ELAVL1 plays an important role in stabilizing the mRNA sequence. Competitive binding to mRNA in the 3′-UTR by ELAVL1 prevents exonuclease- or endonuclease-mediated degradation induced by other RBPs (Chen et al., 1995; Fan and Steitz, 1998b). In one specific case, ELAVL1 promotes neuronal movement by the stabilizing of profilin 1 mRNA (Chen et al., 1995). Similarly, ELAVL1 binding extends the half-life of *CX43* mRNA in rat liver epithelial cells (Ale-Agha et al., 2009).

In addition to interacting with the 3′-UTR, ELAVL1 also has multiple effects in other mRNA regions. For example, ELAVL1 can reduce the activity of the internal ribosome entry site (IRES) in the initiation of translation by binding to the 5′-UTR of the mRNA that codes for the insulin like growth factor type 1 receptor (Meng et al., 2005). Conversely, ELAVL1 can stimulate the initiation of translation of X-linked inhibitor of apoptosis (XIAP) mRNA by binding to the IRES in the 5′-UTR of the XIAP mRNA (Durie et al., 2011).

Other members of the ELAVL protein family have not been studied to the extent that ELAVL1 has, but these other family members have been shown to be important in multiple physiological and pathological processes. ELAVL2 (also known as HuB or Hel-N1), ELAVL3 (HuC) and ELAVL4 (HuD) are mainly distributed in neuronal cells (Ripin et al., 2019). ELAVL2 plays a key role in several processes in the early stages of neuronal differentiation, such as cell cycle exit (Hambardzumyan et al., 2009). ELAVL3 has been shown to affect brain function in that low expression levels of ELAVL3 correlated with impaired spatial learning ability of mice and led to the down-regulation of expression of growth associated protein-43 (Quattrone et al., 2001). In addition, the levels of the ELAVL4 transcript and protein in the superior cervical ganglion were found to decrease after the severing of the axon (Deschenes-Furry et al., 2007). These results show that ELAVL2-4 are involved in the development and functioning of the nervous system. Interestingly, however, the functions of these three protein family members go far beyond the brain. They are also essential in the maintenance of physiological functions and the regulation of the occurrence and development of a variety of diseases (Mazan-Mamczarz et al., 2003; Casolaro et al., 2008; Beauchamp et al., 2010; Ahuja et al., 2016; Lee et al., 2018; Zhao et al., 2019).

## The Regulation of ELAVL Proteins by Non-RNA Molecules

The regulation of ELAVL proteins by non-RNA molecules can be divided into three categories: regulation of protein expression level, regulation of nucleocytoplasmic shuttling, and regulation of the binding of ELAVL proteins to the transcripts in cytoplasm. In gastric tumorigenesis, the activation of AKT serine/threonine kinase promotes the binding of Nuclear Factor-kappa B to the *ELAVL* promoter, which enhances transcription and the stability of the transcripts (Kang et al., 2008). In addition, the esophageal cancer related gene 2 protein has been shown to increase ubiquitination and degradation of ELAVL1 in the colon cancer-derived RKO cell line and the breast cancer-derived MCF7 cell line, but this protein failed to produce similar effects on several non-ubiquitinable mutant forms of ELAVL1 (Lucchesi et al., 2016).

In response to proliferative signals, the phosphorylation of ELAVL1 protein by cyclin-dependent kinase 1 at S202 prevents its translocation to the cytoplasm, resulting in the inhibition of its pro-proliferation and anti-apoptotic effects (Kim et al., 2008). Upon cessation of the external signal, two mechanisms serve to promote relocalization to the cytoplasm. One mechanism involves polyADP-ribosylation of ELAVL1 by poly (ADP-ribose) polymerase 1 (Ke et al., 2017), and a second mechanism is mediated by p38 mitogen-activated protein kinase (Farooq et al., 2009). In another enzyme-controlled mechanism of regulation, sulfhydration by cystathionine γ lyase prevents the homodimerization of ELAVL1, which ultimately leads to decreases in activity to levels insufficient to increase the expression level of downstream genes in mouse endothelial cells (Bibli et al., 2019).

## Interactions Between ELAVL Proteins and Various Classes of RNA Molecules

### Interactions Between ELAVL Proteins and mRNAs

One of the main functions of the ELAVL protein family is to regulate the stability and half-life of downstream mRNA. Therefore, their regulatory effects on cells almost entirely depend on the function of downstream mRNAs and the direction of regulation. The most common binding sites where ELAVL proteins interact with mRNAs, AREs in the 3′-UTR, are found in up to 8% of human genome transcripts (Bakheet et al., 2006). These AREs are often regarded as regulatory elements that promote mRNA decay; most RBPs that bind to this region, such as tristetraprolin, butyrate response factor 1, AU-binding factor 1, and KH-type splicing regulatory protein, greatly reduce the half-life of the target RNA (Gherzi et al., 2004; Lykke-Andersen and Wagner, 2005; Gratacós and Brewer, 2010). In most cases, however, the effect of the binding of ELAVL proteins to a downstream mRNA supports stability (Barreau et al., 2005). Previously discovered mRNAs that have been found to bind to the ELAVL family are shown in Supplementary Table 1.

### Interactions Between ELAVL Proteins and ncRNAs

#### Micro RNA (miRNA)

Various miRNA molecules can bind to the 3′-UTRs of mRNA to decrease the stability of the mRNA. Therefore, the regulation of miRNAs to the ELAVL protein family is mainly reflected in the stability of mRNAs of the latter (Table 1). For example, miR-133, which targets the *ELAVL1* mRNA and is sponged by long intergenic non-protein coding RNA, muscle differentiation 1 (linc-MD1), regulates the expression of ELAVL1. The ELAVL1 protein in turn promotes the interaction between linc-MD1 and miR-133 in the early stages of myogenesis (Legnini et al., 2014). Through targeting AREs in *ELAVL1* mRNA, miR-155-5p negatively regulates the protein level of ELAVL1 and thus the migration of tumor cells in colorectal cancer (Al-Haidari et al., 2018). In breast cancer, miR-125a inhibited cell proliferation and promoted apoptosis by downregulating ELAVL1 which was highly expressed in cancer cells, and this effect was partially rescued by ELAVL1 overexpression (Guo et al., 2009). In normal human dermal fibroblasts, the overexpression of miR-520d-5p has been shown to down-regulate ELAVL2 and restore cell proliferation; down-regulation of ELAVL2 with small interfering RNA alone achieved the same effect (Ishihara et al., 2014). In mutant motor neurons, decreased expression of miR-375 resulted in increased expression of its downstream targets, which include ELAVL4 as well as p53. These changes promoted the apoptosis and fragility of mutant motor neurons in amyotrophic lateral sclerosis (De Santis et al., 2017).

**TABLE 1:** MiRNAs interacting with ELAVL proteins.

Member of protein family	miRNA	Interaction and effect	PMID
ELAVL1	miR-199a	Prevent pre-miR-199a from maturing	26346275
	miR-27	Competitively bind downstream mRNA	25533351
	miR-133	Inhibit *ELAVL1* mRNA	24440503
	miR- 155-5p		29471005
	miR-125a		19875930
	miR-519		19088191
	miR-582-3p		32600329
	miR-291b-3p		30106126
	miR-326		32968928
	miR-514a-5p		32370736
	miR-3127-5p		30317610
		miR-23c		27964927
		miR-146b-5p		27166258
ELAVL2	miR-520d	Inhibit *ELAVL2* mRNA	25303886
ELAVL4	miR-375	Inhibit *ELAVL4* mRNA	28988989
	miR-129-5p		32335272

In turn, ELAVL proteins can regulate miRNAs maturation or co-regulate downstream with miRNAs. In another mode of regulation for miR-199a, hypoxia-induced expression of ELAVL1 prevents the maturation of pre-miR-199a, thereby promoting enhancement of glycolysis through impacts of miR-199a on hexokinase 2 and pyruvate kinase 2 expression in the tumor microenvironment (Zhang et al., 2015). It has been shown that miR-27 targets the mRNA encoding zinc finger protein 36 mRNA in macrophages, but ELAVL1 and miR-27 compete for binding to the 3′-UTR of this mRNA to regulate its stability (Lu et al., 2014). These examples indicate that there is significant crosstalk among RBPs, miRNAs and mRNAs, and that the regulation is not limited to a few isolated cases.

#### Long Non-coding RNA (lncRNA)

The mechanisms by which lncRNAs regulate mRNA activity through the ELAVL protein family, especially ELAVL1, include 1) direct binding to ELAVL1 and promotion of its binding to downstream mRNA, 2) inhibiting of the expression of ELAVL1, 3) stabilizing ELAVL1 protein, 4) direct binding to ELAVL1 and blocking of its binding to downstream mRNA, and 5) promoting the translocation of ELAVL1 from the nucleus to the cytoplasm (Table 2).

**TABLE 2:** LncRNAs that regulate ELAVL proteins.

Member of protein family	LncRNA	Interaction and effect	PMID
ELAVL1	LINC00707	Binds to ELAVL1 and stabilizes downstream mRNA	30502359
	RMST		31636039
	B4GALT1-AS1		30182452
	MIR100HG		30102375
	lincRNA-UFC1		25449213
	HMS		34302808
	lAK136714		34015766
	AGAP2-AS1		33273726
	TUG1		33047284
	TSLNC8		32951177
	ZEB1-AS1		31922280
	HOXB-AS1		31886581
	SNHG7		31026094
	EGFR-AS1		30770799
	LINC00707		30502359
	LINC00324		29915327
	SPRY4-IT1		27853262
	APOA4-AS		27131369
	lncRNA OCC-1	Inhibit the expression of ELAVL1	29931370
	ASB16-AS1		33219221
	AK058003		28035067
	FAM83H-AS1	Stabilize ELAVL1	30831080
	CAAlnc1	Blocks ELAVL1 from binding downstream	30807648
	FENDRR		31180580
	OIP5-AS1		26819413
	OSER1-AS1		33113263
	RPSAP52		31831098
	tie1AS		29724820
	MALAT1		27197265
		lncRNA MAARS	Binds to ELAVL1 and promotes its translocation to the cytoplasm	33262333

First, LINC00707 has been reported as a malignant factor in the progression of lung adenocarcinoma and gastric cancer. LINC00707, which is highly expressed in tumor tissues, has been shown to form a complex with ELAVL1 protein. This complex increases the expression of downstream proteins, such as vav guanine nucleotide exchange factor 3/F11 receptor, and ultimately leads to tumor progression and a poorer tumor prognosis (Xie et al., 2019). In a similar way, lncRNA RMST enhances the binding of ELAVL1 to the mRNA of the target gene *DNMT3B*, thereby increasing the expression of *DNMT3B* and global levels of DNA methylation (Peng et al., 2020).

Second, ELAVL1 interacts with lncRNA OCC-1, which acts as a protective factor in colorectal cancer, inhibits the growth of tumor cells *in vivo* and *in vitro*. This inhibition is achieved by sensitizing ELAVL1 to ubiquitination and making it prone to degradation (Lan et al., 2018). In the third type of regulation, up-regulated FAM83H-AS1 binds to ELAVL1 and stabilizes it, which can induce cell metastasis and resistance to radiotherapy in ovarian cancer (Dou et al., 2019). As described in the fourth mode of regulation, pull-down assays and RNA immunoprecipitation have confirmed the binding relationship between CAAlnc1 and ELAVL1, which blocked the binding of ELAVL1 to mRNAs associated with fat production (Shen et al., 2019).

According to multiple reports, lncRNA MAARS, which is positively correlated with the progression of atherosclerosis disease, interacts with ELAVL1 and reduces its cytoplasmic localization, which reduces the apoptosis of macrophages and delays the course of the disease (Simion et al., 2020).

#### Circular RNA (circRNA)

The class of circRNAs includes closed circular ncRNA molecules that are not easily degraded and have been shown to regulate the progression of various diseases (Li et al., 2015). For example, circRHOBTB3 is expressed at relatively low levels in hepatocellular carcinoma, and it has been reported that over-expression of circRHOBTB3 can lead to degradation of ELAVL1 and thus inhibition of the expression of the ELAVL1 target gene *PTBP1* (Chen et al., 2021b). ELAVL1 also interacts competitively with circDLC1 to negatively regulate the expression of the gene encoding matrix metalloproteinase 1 (Liu et al., 2021); this mechanism occurs in a similar manner with regard to circPPM1F and its target gene *PPM1F* (Zhang et al., 2020) and circPABPN1 and its target gene *ATG16L1* (Li et al., 2020).

**FIGURE 2:**
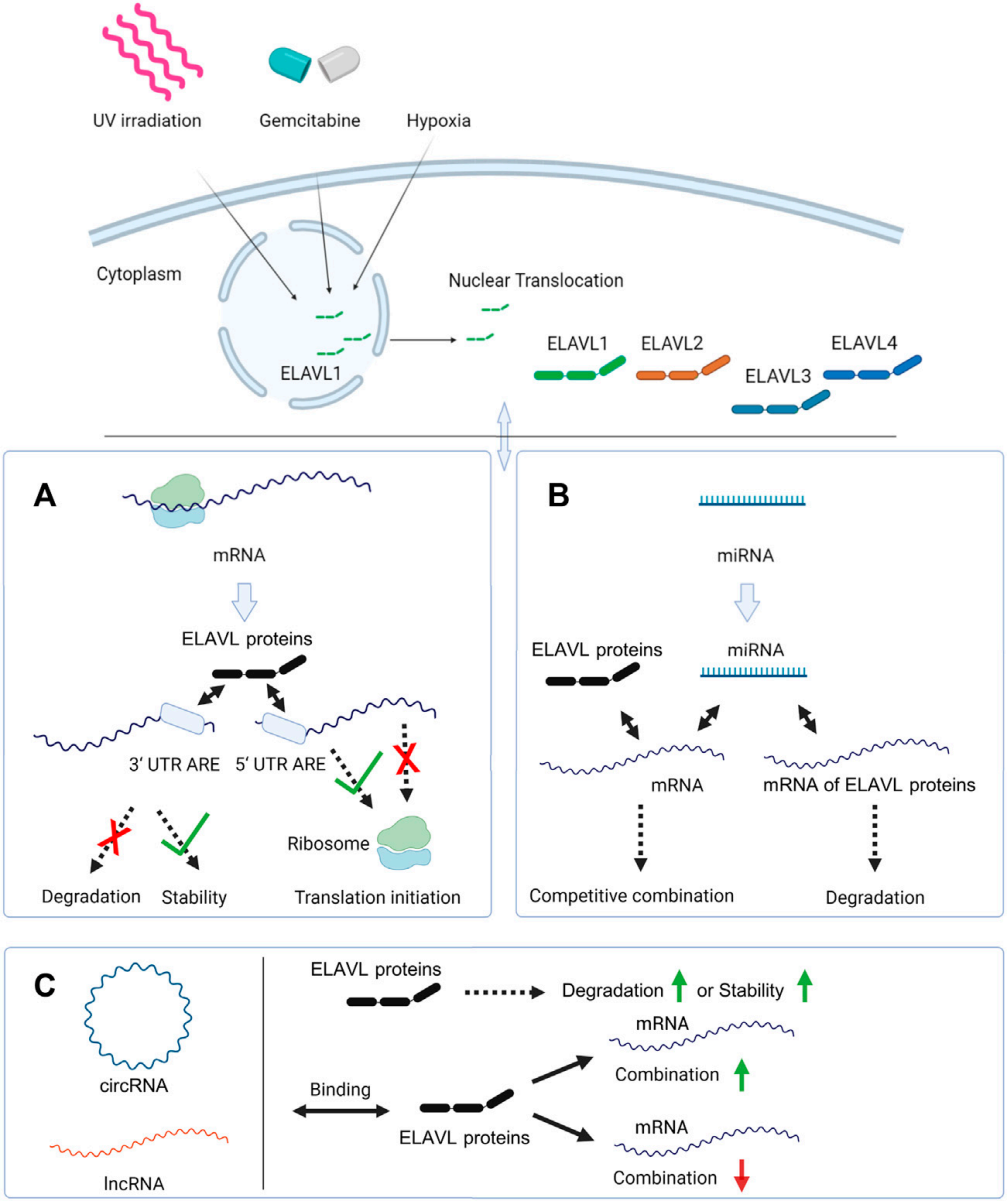
The interaction of ELAVL proteins with mRNAs and ncRNAs. In response to external stimuli like UV irradiation, gemcitabine or hypoxia, ELAVL1 is transported from the nucleus to the cytoplasm. And ELAVL2-4 are mainly localized in the cytoplasm. **(A)** ELAVL proteins inhibit mRNAs’ degradation and stabilize them by binding the 3′-UTR. In another regulation mode, ELAVL proteins can promote or inhibit mRNAs’ translation by binding the 5′-UTR; **(B)** miRNAs and ELAVL proteins co-regulate downstream mRNAs, and some miRNAs can regulate the expression of ELAVL proteins by binding their mRNAs. **(C)** circRNA and lncRNAs promote ELAVL proteins’ degradation or stabilize them by binding to them. And circRNAs and lncRNAs can also promote or inhibit the binding of ELAVL proteins to downstream mRNAs. Created with BioRender.com.

The complex between circ-CCND1 and ELAVL1 promotes the expression of the gene encoding cyclin D1 and ultimately leads to the malignant proliferation of laryngeal squamous cell carcinoma (Zang et al., 2020). A similar mechanism has been found in the relationship among CircAGO2/ELAVL/*AGO2* (Chen et al., 2019). Further studies have shown that circRNA-mediated positive regulation of downstream mRNAs may be achieved by promoting the cytoplasmic relocation of ELAVL1; thus, circBACH1 binds directly to ELAVL1 and mediates its translocation from the nucleus, thereby increasing its binding to the mRNA encoding p27 (Liu et al., 2020b). At the level of transcription, nuclear-localized circ-HuR derived from *ELAVL1* can bind to the transcription factor cellular nucleic acid-binding protein to block this protein from binding to the *ELAVL1* promoter, resulting in a decreased expression of ELAVL1 (Yang et al., 2019). In another regulatory mechanism, direct binding of Hsa_circ_00074854 to the ELAVL1 protein improves the stability of the protein, and ultimately promotes hepatocellular carcinoma migration, invasion and epithelial-mesenchymal transition (Wang et al., 2021). The interaction between ncRNA and ELAVL proteins is shown in Figure 2.

## ELAVL Proteins in Pathological and Physiological Processes

### ELAVL Proteins in Cancers

#### ELAVL1

**FIGURE 3:**
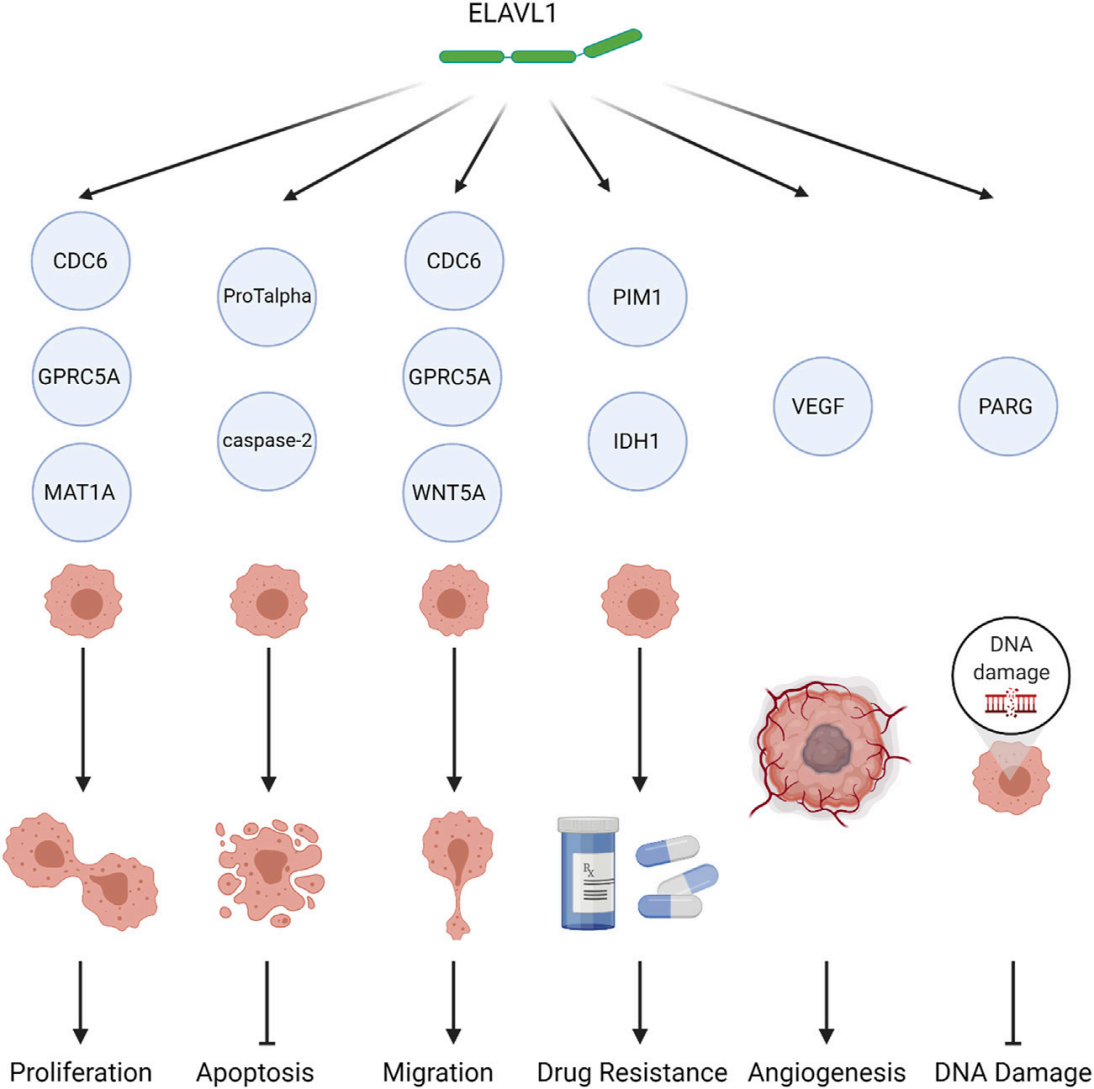
The role of ELAVL1 in tumors. ELAVL1 affects tumor biology by either (1) promoting proliferation, (2) inhibiting apoptosis, (3) promoting migration, (4) inducing drug resistance, (5) promoting angiogenesis, or (6) reducing DNA damage. Created with BioRender.com.

ELAVL1 is indispensable to life. For example, the *ELAVL1* gene is expressed during mouse embryonic development and growth cycle (Gouble and Morello, 2000), and the knockout of mouse *ELAVL1* leads to hematopoietic failure, loss of intestinal villi, and death within 10 days (Ghosh et al., 2009). Thus, ELAVL1 protein is necessary in the maintenance of normal life processes. On the other hand, dysregulation of the expression of the *ELAVL1* gene or the activity of the protein can also lead to aberrant cellular growth and cancer. A schematic of the relationships between ELAVL1 and cancer is shown in Figure 3.

##### Colorectal Cancer

The functions of ELAVL1 in colorectal cancer have been studied extensively. Importantly, studies have connected ELAVL1 to cyclooxygenase 2 (COX-2), which has been shown through *in vivo* and *in vitro* studies to be a key factor in the malignant progression of colorectal cancer. As noted, ELAVL1 typically distributes mainly to the nucleus, but β-catenin can promote the cytoplasmic translocation of ELAVL1 (Lee and Jeong, 2006). Enrichment of ELAVL1 in the cytoplasm prolongs stabilization of the mRNA that encodes for COX-2 by binding with an ARE in the 3′-UTR, causing an increase in COX-2 protein levels (Dixon et al., 2001). Accordingly, the cytoplasmic localization of ELAVL1 has been shown to be significantly positively correlated with tumor stage (Denkert et al., 2006).

Interactions with the cell cycle also explain involvement of ELAVL1 in colorectal cancer. For example, ELAVL1 positively regulates the cell division 6 protein, which is highly expressed in colorectal cancer and which drives both the malignant behavior of colorectal cancer and its resistance to oxaliplatin (Cai et al., 2019). It has been reported that in the colorectal carcinoma RKO cell line, the levels of ELAVL1 in the cytoplasm increase during the late G_1_, S, and G_2_ phases of the cell cycle, and it binds to the 3′-UTR of mRNAs encoding cyclins A and B1, resulting in enhanced stability of these mRNAs and increased expression of the proteins, ultimately leading to increased cell proliferation (Wang et al., 2000). In animal experiments, overexpressing ELAVL1 in RKO cells results in increased tumor sizes upon injection into nude mice (de Silanes et al., 2003).

##### Ovarian Cancer

ELAVL1 can promote the expression level of COX-2 in ovarian cancer as well as in colorectal cancer, and COX-2 is also positively correlated with poor prognosis and high-grade of ovarian cancer. When the nuclear translocation of ELAVL1 is suppressed, the expression of COX-2 decreases *in vitro* (Erkinheimo et al., 2003). Accordingly, it has been found that levels of ELAVL1 in the cytoplasm of cells in ovarian cancer tissues was significantly increased relative to levels in borderline tumors or normal ovaries (Denkert et al., 2004). Another interaction with ovarian cancer involves miR-519, which targets the 3′-UTR of *ELAVL1* mRNA, inhibiting its translation and slowing cell division of A2780 cells, a human ovarian cancer cell line, *in vitro* (Abdelmohsen et al., 2008). Also, in A2780 cells, glucose deprivation has been shown to enhance ELAVL1-dependent *TUBB3* expression at the mRNA and protein levels, resulting in tumor invasion (Raspaglio et al., 2010).

##### Breast Cancer

ELAVL1 affects the development of breast cancer by regulating the mRNAs associated with a variety of proteins. Accordingly, among familial non-BRCA1/2 breast cancer patients, ELAVL1 can be used as an independent prognostic factor, associated with low survival rate and high tumor malignancy (Heinonen et al., 2007). Mechanistically, ELAVL1 promotes the expression of interleukin (IL)-8, which has clear connections with the progression of breast cancer, *via* binding to the 3′-UTR of the mRNA encoding IL-1β (Suswam et al., 2005). In addition, the abnormal expression of cyclin E1, Wnt-5a, thrombospondin 1 and the colony stimulating factor receptor is directly related to the increased expression of ELAVL1 in breast cancer models (Guo and Hartley, 2006; Leandersson et al., 2006; Mazan-Mamczarz et al., 2008; Woo et al., 2009). When ELAVL1 is silenced, the programmed death of tumor cells increases and invasion is inhibited (Heinonen et al., 2011). ELAVL1 is also regulated by upstream factors in breast cancer. Levels of miR-125 are negatively correlated with ELAVL1; miR-125 may thus act as an inhibitor of ELAVL1 to decrease translation by binding with its mRNA (Guo et al., 2009).

##### Pancreatic Cancer

The role of ELAVL1 in pancreatic cancer remain controversial. Overexpression of ELAVL1 in pancreatic cancer cells has been shown to increase the sensitivity of patients to gemcitabine treatment. The mechanism of this effect involves the binding of ELAVL1 to and the promotion effect of the mRNA that encodes deoxycytidine kinase whose products can activate gemcitabine (Costantino et al., 2009). In another report, ELAVL1 was also shown to be involved in the apoptosis of pancreatic cancer cells exposed to gemcitabine. ELAVL1 translocates to the cytoplasm after gemcitabine treatment, where it binds to the mRNA of retinoic acid-induced protein 3, which acts as an oncogene, leading to an increase of it at the initial stage of drug treatment (Zhou et al., 2016). Hypoxia in the tumor microenvironment can similarly induce nucleocytoplasmic shuttling of ELAVL1, which then promotes the expression of the PIM1 serine/threonine kinase, which leads to resistance to oxaliplatin (Blanco et al., 2016). In pancreatic ductal adenocarcinoma, ELAVL1 promotes the translation of poly (ADP-ribose) glycohydrolase mRNA, leading to enhanced DNA repair and resistance to the PARP inhibitor olaparib (Chand et al., 2017).

##### Other Types of Cancer

ELAVL1 has been found to play important roles in multiple kinds of tumors. For instance, ELAVL1 has been found to be highly expressed in prostate cancer, and it thus acts as an independent predictor positively correlating with tumor staging and metastasis. ELAVL1 promotes cell proliferation and migration of cells of the prostate cancer lines LNCaP and PC-3 by targeting vascular endothelial growth factors A and C and COX-2 (Barbisan et al., 2009; Mitsunari et al., 2016).

In hepatocellular carcinoma cells, the highly expressed lincRNA-UFC1 directly binds to ELAVL1, leading to an increase of β-catenin mRNA and protein and finally increased cell proliferation and decreased apoptosis (Cao et al., 2015). Similarly, hsa_circ_0074854 promotes the migration and invasion of hepatocellular carcinoma cells by stabilizing ELAVL1 (Wang et al., 2021). On another hand, ELAVL1 binds pre-miRNA-199a to prevent its maturation, leading to enhanced glycolytic metabolism in hepatocellular carcinoma cells in response to hypoxia (Zhang et al., 2015).

In glioblastoma multiforme and adjacent tissues, high expression of ELAVL1 can also be detected (Nabors et al., 2001). In glioblastoma, pyruvate kinase M2, which is up-regulated, binds to ELAVL1 and promotes its cytoplasmic localization, prompting tumor cells to enter a dividing state and promoting cell growth (Mukherjee et al., 2016). In a nude mouse model of glioblastoma, knockdown of *ELAVL1* reduced tumor growth and proliferation, and prolonged survival time (Wang et al., 2019b).

Generally speaking, ELAVL1 often appears as a malignant factor. On the one hand, it is indispensable in life activities. On the other hand, the tumorigenic effects of high expression of ELAVL1 acts as an important contributor to the progression and invasion of many types of tumor through various pathways. Therefore, ELAVL1 may be a potential drug target with universal applicability.

#### ELAVL2-4

The three other family members, ELAVL2, ELAVL3 and ELAVL4, initially received much attention as neuroendocrine markers for small cell lung cancer (SCLC) (King, 1997; D'Alessandro et al., 2008). Among them, ELAVL4 received the most attention in SCLC, because it was found to be expressed in 100% of SCLC cells and more than 50% of neuroblastoma cells, and treatment targeting ELAVL4 can reduce tumor progression in nude mouse models (Ohwada et al., 1999; Ehrlich et al., 2014). As the antigen target of autoreactive CD4^+^ T cells, ELAVL4 may directly participate in cell-mediated anti-tumor immunity and nervous system damage (Benyahia et al., 1999).

At the level of post-transcriptional regulation, ELAVL4 regulates RNA as an RBP, but in tumorigenic neuroblasts, ELAVL4 also takes part in the nuclear processing and stability of the pre-mRNA of the proto-oncogenic transcription factor N-myc (Lazarova et al., 1999). ELAVL4 has also been shown to interact with both the 3′-UTR and 5′-UTR regions of the p27 mRNA to promote its translation leading to tumor suppression, but ELAVL4 and p27 levels are both reduced in pancreatic neuroendocrine tumors (Kim et al., 2018). Outside of SCLC, ELAVL2 has been shown to be an independent risk factor in esophageal squamous cell carcinoma, and it increases the resistance of these tumor cells to paclitaxel and cisplatin (Zhao et al., 2019).

There are also notable cancer-related interactions between members of the ELAVL family. A combination of ELAVL2 and ELAVL1 has been shown to localize to the nucleus and to be indispensable in the activation of several proto-oncogenes, including v-fos, v-ets, and v-myc (Hatanaka et al., 2019). Also, by binding with a structure containing an AU-rich sequence, ELAVL2 and ELAVL4 together inhibit the assembly of the core complex of telomerase to reduce its activity and cell growth in human neuroblastoma cells; notably, the activity of this complex antagonizes the function of ELAVL1 (Cheng et al., 2021). Surprisingly, considering the importance of the other ELAVL family members, few reports link ELAVL3 to tumor development or progression.

### ELAVL Proteins in Disorders of the Nervous System

The ELAVL protein family was originally best known for its associations with the nervous system (Akamatsu et al., 1999). In the development of the neocortex, the deletion of ELAVL1 reduces the phosphorylation of eIF2a and eEF2 and the formation of polysomes, ultimately leading to the mis-localization of mRNAs. The lack of ELAVL1 reduces the stability of *PFN1* mRNA and affects actin polymerization, resulting in the mis-localization of neurons in the neocortex (Kraushar et al., 2014; Zhao et al., 2020a). It also participates in cellular metabolism and protection from oxidation-induced neurodegeneration (Skliris et al., 2015).

With regard to various disease states, ELAVL1 has either protective or damaging effects, depending on the circumstances. In spinal muscle atrophy, ELAVL1 stabilizes survival motor neuron transcripts, which leads to accumulation of the protein products, thus alleviating the loss of alpha motor neurons that otherwise lead to progressive muscle atrophy (Farooq et al., 2009). In the occurrence and progress of neurodegenerative diseases, including age-related macular degeneration, ELAVL1 promotes the early elevation and accumulation of P62 in response to the early activation of autophagy, clearing protein multimers and alleviating neurodegenerative effects (Marchesi et al., 20182018).

On the other hand, ELAVL1 has been found to play deleterious roles in Huntington’s disease and amyotrophic lateral sclerosis. In Huntington’s disease, which is caused by mutations in the *HTT* gene and abnormal accumulation of the HTT protein, the HTT protein itself induces ELAVL1 to stabilize *HTT* mRNA, forming a positive feedback loop (Zhao et al., 2020b). Moreover, inhibition of ELAVL1 has been shown to block the chronic activation of microglia in amyotrophic lateral sclerosis and to delay the course of this disease (Matsye et al., 2017).

In neurons, ELAVL2, ELAVL3, and ELAVL4 function in stages. ELAVL2 protein is expressed in early neuron progenitor cells through to mature neurons, while ELAVL3 and 4 are expressed later than ELAVL2 and function mainly in cortical neuron development (Yano et al., 2016). Multiple studies have linked ELAVL4 to neuron development and plasticity (Bronicki et al., 2012; Loffreda et al., 2020). ELAVL4 is engaged in stabilization of tau microtubule-associated protein transcripts and maintains axon development in neuronal cells. Accordingly, the inhibition of ELAVL4 results in the cessation of axonal growth (Aranda-Abreu et al., 1999; Fukao et al., 2009; Hao le et al., 2017), and a deficiency of ELAVL4 in mice leads to transient impaired cranial nerve development during the embryonic period *in vivo* (Akamatsu et al., 2005).

Similar to ELAVL1, ELAVL4 is indispensable for establishing neocortex and hippocampal circuits and maintaining the function of these circuits (DeBoer et al., 2014). Furthermore, in the adult subventricular zone neural stem and progenitor cells, ELAVL4 promotes neuronal differentiation through special AT-rich sequence-binding protein 1 (SATB1). A lack of SATB1 affects the maturation of neuronal stem cells, and the overexpression of SATB1 in ELAVL4-suppressed cells can restore the neuronal differentiation phenotype (Wang et al., 2015). ELAVL2 promotes the exit from the cell cycle during the neuronal stem cell maturation, and overexpression of ELAVL2 restricts the proliferation of neuronal stem cells (Hambardzumyan et al., 2009). For ELAVL3, a low level of ELAVL3 leads to the impairment of spatial learning ability of mice with lowered expression of growth-associated protein 43 (Quattrone et al., 2001).

### ELAVL Proteins in Other Physiological and Pathological Process

**FIGURE 4:**
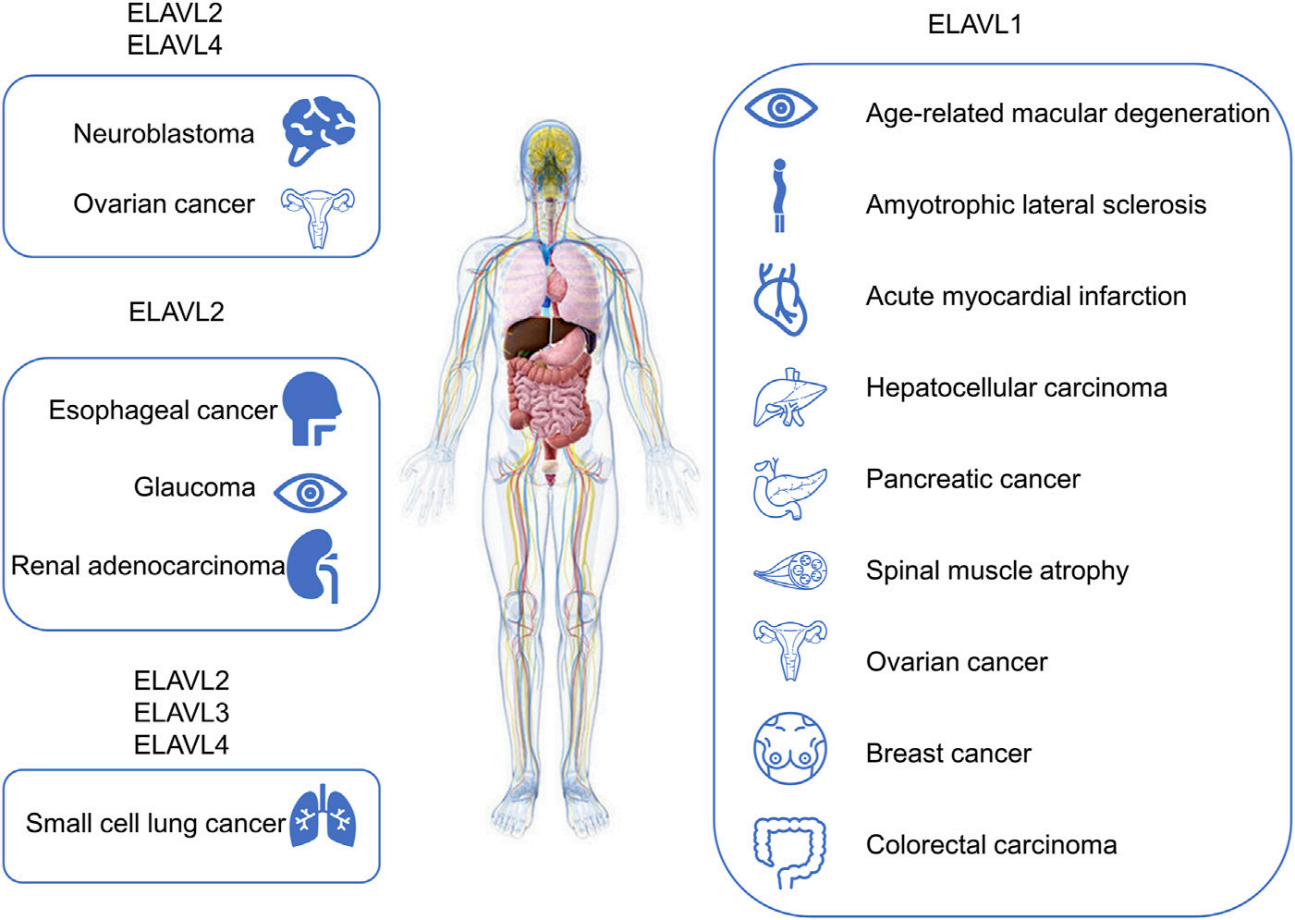
Diseases or pathophysiological processes related to ELAVL proteins. Diseases related to ELAVL proteins are included in the figure.

In addition to cancer and processes involving the nervous system, the ELAVL protein family is also involved in muscle differentiation (Beauchamp et al., 2010), aging (Lee et al., 2018), inflammation (Casolaro et al., 2008), stress events (Mazan-Mamczarz et al., 2003; Ahuja et al., 2016) and other processes. Figure 4 shows how tumors and other different diseases interact with ELAVL proteins.

The impact on muscle differentiation involves a novel proteolytic cleavage of ELAVL1. When transferred to the cytoplasm in myoblasts, some ELAVL1 is cleaved into two fragments, cleavage products (CP) 1 and 2, of sizes 24 and 8 kDa, respectively. CP1 forms a complex with ELAVL1 import factor transportin-2 (TRN2), allowing uncleaved ELAVL1 to remain in the cytoplasm. The other fragment, CP2, promotes myogenesis (Beauchamp et al., 2010).

The known impact of ELAVL1 on aging involves interactions with the telomeric protein TIN2. ELAVL1 binding destabilizes *TIN2* mRNA to decay quickly. Therefore, when ELAVL1 is inhibited, the expression of TIN2 protein in the mitochondria are increased. These factors correlate with increased levels of ROS, ultimately leading to cell senescence (Lee et al., 2018).

Inflammatory stimuli lead to poly ADP-ribosylation of D226 of ELAVL1. Modified ELAVL1 oligomerizes in the presence of PARP1, resulting in the protection of pro-inflammatory mRNA from degradation induced by miRNA and other factors. Therefore, treatments targeting ELAVL1 alleviate the lipopolysaccharide-induced accumulation of inflammatory cells in the airways of mice (Ke et al., 2021). In another inflammatory disease, pterygium, the activation of matrix metalloproteinase 9 by ELAVL1 amplifies the pro-inflammatory effect of IL-1β (Cui et al., 2020). The combination of ELAVL1 and matrix metalloproteinase 9 also appears in the inflammatory infiltration of cardiomyocytes in acute myocardial infarction. IL-10 can inhibit this combined effect to alleviate fibrosis and inflammation, and ultimately reduce damage to left ventricular function (Krishnamurthy et al., 2009).

Under the influence of different stressors, the function of ELAVL1 changes accordingly. Low-level UV irradiation induces the translocation of ELAVL1 to the cytoplasm, where it interacts with and preserves transcription products by binding with them after the formation of DNA damage. At the same time, the transcription process is inhibited to prevent the generation of false transcripts. In this mode, ELAVL1 is beneficial to cell survival (Mazan-Mamczarz et al., 2003). On the other hand, after ionizing radiation, ELAVL1 is separated from almost all mRNAs, including proliferation-related and apoptosis-related proteins. While this mechanism is different from that associated with UV irradiation, it similarly contributes to a better survival outcome for cells (Masuda et al., 2011). However, when cells are subjected to high pressure stress, ELAVL1 is translocated to the cytoplasm and where it is cleaved by caspases at A226, and this action amplifies apoptotic signals (Mazroui et al., 2008).

## Inhibitors of ELAVL1

**FIGURE 5:**
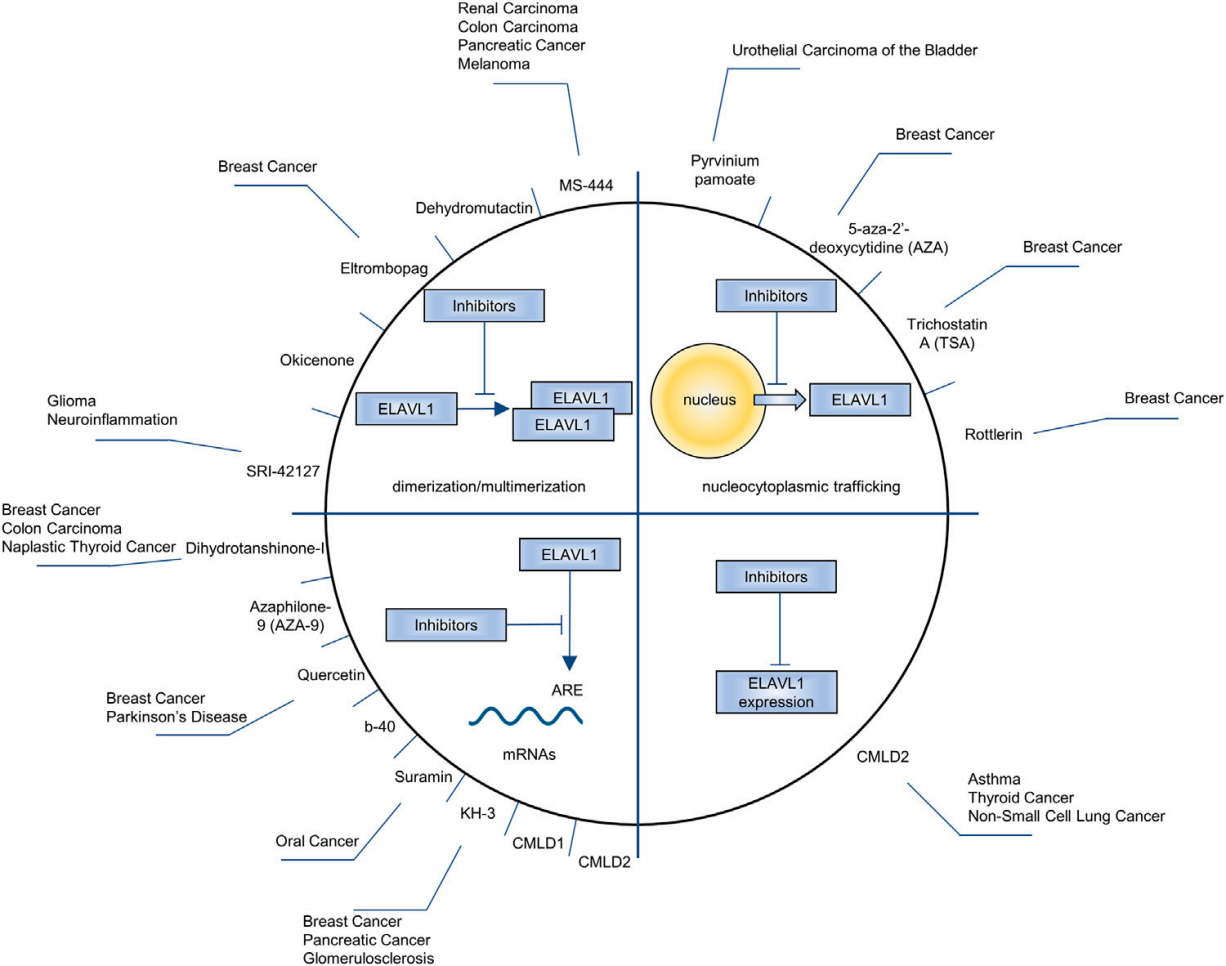
The drugs being used to inhibit ELAVL1, the processes how drugs affect ELAVL1 and the diseases the drugs aim to alleviate.

Research on the inhibitors of ELAVL proteins has mainly focused on ELAVL1. In view of the positive regulation of ELAVL1 in tumor promotion, the research and development of inhibitors is of significance. At present, the effects of inhibitors on ELAVL1 are mainly focused in four directions: inhibiting its nucleocytoplasmic trafficking, blocking its binding to mRNA, suppressing its dimerization/multimerization and downregulating its expression. As shown in Figure 5, MS-444 (Meisner et al., 2007), dehydromutactin (Meisner et al., 2007), okicenone (Meisner et al., 2007), eltrombopag (Zhu et al., 2020) and SRI-42127 (Filippova et al., 2021) inhibit the dimerization/multimerization of ELAVL1; 5-aza-2′-deoxycytidine (AZA) (Hostetter et al., 2008), trichostatin A (TSA) (Hostetter et al., 2008), pyrvinium pamoate (Guo et al., 2016) and Rottlerin (Latorre et al., 2012) inhibite ELAVL1’s nucleocytoplasmic trafficking; Dihydrotanshinone-I (Lal et al., 2017), azaphilone-9 (AZA-9) (Kaur et al., 2017), quercetin (Chae et al., 2009), b-40 (Chae et al., 2009), suramin (Kakuguchi et al., 2018), KH-3 (Wu et al., 2020) and CMLD1 (Wu et al., 2015) block ELAVL1’s binding to mRNA; CMLD2 downregulates ELAVL1’s expression (Muralidharan et al., 2017) and blocks its binding to mRNA (Wu et al., 2015). Among these inhibitors, MS-444, dehydromutactin, okicenone, SRI-42127, AZA-9, b-40, KH-3, CMLD1 and CMLD2 are specific inhibitors of ELAVL1, while others are not. For example, eltrombopag often acts as a thrombopoietin (TPO) receptor agonist (Bussel et al., 2019), AZA as a DNA methyltransferase inhibitor (Song et al., 2022) and TSA as a histone deacetylase inhibitor (He et al., 2022). Pyrvinium pamoate (Faheem et al., 2022), Rottlerin (Hufnagel et al., 2009), dihydrotanshinone-I (Sun et al., 2022), quercetin (Zaragozá et al., 2022) and suramin (Zhang et al., 2022) all have effects on other physiological or pathological processes.

## Conclusion and Perspective

This review summarizes the role of ELAVL proteins in various pathophysiological processes and in regulating mRNA and ncRNA. Given the ubiquity of ELAVL proteins existence and their role in a variety of diseases, it is logical to develop innovative small molecules. Based on the interaction mechanism between ncRNA and ELAVL proteins, the development of ncRNA analogs to promote the degradation of ELAVL proteins or inhibit the translation of their mRNAs is worth discussing. Developing chemically inactive analogs of ELAVL proteins to interfere with their synergy or competition with ncRNA in a competitive manner may also provide a new perspective to reduce the functionality of the protein family. Notably, although the ELAVL proteins have promising potential as a therapeutic target, many questions still need to be further explored. Interfering with ELAVL proteins seems to be a new strategy; however, considering that they are an integral part of life activities and interact with too many RNA molecules, whether their intervention will cause other unexpected side effects needs to be handled carefully. Is it better to use it alone or in combination with other drugs? According to previous reports, inhibition of ELAVL1 sensitized tumors to treatment with platinum-based drugs, including oxaliplatin and cisplatin (Young et al., 2009). What about drugs other than platinum-based drugs? In addition, considering ELAVL2-4 participating in many pathological processes and the similarity of the structure between ELAVL2-4 and ELAVL1, substantial study is imminent to fill the lacunae in the development of ELAVL2-4 inhibitors.

## Glossary

AGAP2: ArfGAP with GTPase domain, ankyrin repeat and PH domain 2
AGAP2-AS1: AGAP2 antisense RNA 1
AGO2: Argonaute 2
APOA4-AS: Rho GTPase activating protein 21
ARE: AU-rich element
ASB16: ankyrin repeat and SOCS box containing 16
ASB16-AS1: ASB16 antisense RNA 1
ATG16L1: Autophagy Related 16 Like 1
B4GALT1-AS1: B4GALT1 Antisense RNA 1
BRCA: breast cancer;
CAAlnc1: cachexia-related anti-adipogenesis lncRNA 1
CircAGO2: circular RNA Argonaute 2
circBACH1: circular RNA BTB Domain and CNC Homolog 1
circ-CCND1: circular RNA Cyclin D1
circDLC1: circular RNA DLC1
circ-HuR: circular RNA ELAV Like RNA Binding Protein 1
circRHOBTB3: circular RNA RHOBTB3
circRNA: circular RNA
COX-2: cyclooxygenase 2
CP: cleavage products
CX43: Connexin 43
DNMT3B: DNA methyltransferase 3B
eEF2: Eukaryotic Translation Elongation Fa
EGFR: epidermal growth factor receptor
EGFR-AS1: EGFR antisense RNA 1
eIF2a: Eukaryotic Translation Initiation Factor 2A
ELAVL proteins: Embryonic lethal abnormal vision-like proteins
FAM83H-AS1: family with sequence similarity 83 member H antisense RNA 1
FENDRR: FOXF1 adjacent non-coding developmental regulatory RNA
HCT116: human colon cancer cells 116
Hel-N1: human elav-like neuronal protein 1
HMS: HOXC10 mRNA stabilizing factor
HOXB: homeobox B cluster
HOXB-AS1: HOXB cluster antisense RNA 1
HTT: Huntingtin
HuB: human antigen B
HuC: human antigen C
HuD: human antigen D
HuR: human antigen R
IL: interleukin
IRES: internal ribosome entry site
lincRNA-UFC1: long intergenic noncoding RNA UFC1
lncRNA: Long non-coding RNA
MAARS: Macrophage-Associated Atherosclerosis
MALAT1: metastasis associated lung adenocarcinoma transcript 1
MD1: muscle differentiation 1
MIR100HG: mir-100-let-7a-2-mir-125b-1 cluster host gene
miRNA: micro RNA
mRNA: message RNA
ncRNA: non-coding RNA
NF-κB: Nuclear Factor-kappa B
OCC-1: Overexpressed in colon carcinoma-1
OIP5: Opa interacting protein 5
OIP5-AS1: OIP5 antisense RNA 1
OSER1: oxidative stress responsive serine rich 1
OSER1-AS1: OSER1 antisense RNA 1
p27: protein 27
p53: protein 53
P62: protein 62
PARP1: poly(ADP-ribose) polymerase 1
PFN1: profilin 1
PIM1: Pim-1 Proto-Oncogene, Serine/Threonine Kinase
PPM1F: Protein Phosphatase, Mg2+/Mn2+ Dependent 1F
PTBP1: Polypyrimidine Tract Binding Protein 1
RBP: RNA-binding protein
RMST: habdomyosarcoma 2-Associated Transcript
ROS: reactive oxygen species
RPSAP52: ribosomal protein SA pseudogene 52
RRM: RNA recognition motif
SATB1: sequence-binding protein 1
SCLC: small cell lung cancer
SNHG7: small nucleolar RNA host gene 7
SPRY4: sprouty RTK signaling antagonist 4
SPRY4-IT1: SPRY4 intronic transcript 1
tie1AS: tie1 locus-tie1 antisense
TIN2: TRF1-interacting nuclear protein 2
TRN2: transportin-2
TSLNC8: Tumor suppressor long noncoding RNA on chromosome 8p12
TUBB3: Tubulin Beta 3 Class III
TUG1: aurine up-regulated 1
UTR: untranslated region
UV: Ultraviolet Rays
Wnt-5a: Wnt Family Member 5A
XIAP: X-linked inhibitor of apoptosis
ZEB1: zinc finger E-box binding homeobox 1
ZEB1-AS1: ZEB1 antisense RNA 1.

## References

[B1] Abdelmohsen K. Srikantan S. Kuwano Y. Gorospe M. (2008). miR-519 Reduces Cell Proliferation by Lowering RNA-Binding Protein HuR Levels. Proc. Natl. Acad. Sci. U.S.A. 105, 20297–20302. 10.1073/pnas.0809376106 19088191 PMC2629338

[B2] Ahuja D. Goyal A. Ray P. S. (2016). Interplay between RNA-Binding Protein HuR and microRNA-125b Regulates P53 mRNA Translation in Response to Genotoxic Stress. RNA Biol. 13, 1152–1165. 10.1080/15476286.2016.1229734 27592685 PMC5100343

[B3] Akamatsu W. Fujihara H. Mitsuhashi T. Yano M. Shibata S. Hayakawa Y. (2005). The RNA-Binding Protein HuD Regulates Neuronal Cell Identity and Maturation. Proc. Natl. Acad. Sci. U.S.A. 102, 4625–4630. 10.1073/pnas.0407523102 15764704 PMC555491

[B4] Akamatsu W. Okano H. J. Osumi N. Inoue T. Nakamura S. Sakakibara S.-I. (1999). Mammalian ELAV-like Neuronal RNA-Binding Proteins HuB and HuC Promote Neuronal Development in Both the central and the Peripheral Nervous Systems. Proc. Natl. Acad. Sci. 96, 9885–9890. 10.1073/pnas.96.17.9885 10449789 PMC22305

[B5] Al-Haidari A. Algaber A. Madhi R. Syk I. Thorlacius H. (2018). MiR-155-5p Controls colon Cancer Cell Migration via post-transcriptional Regulation of Human Antigen R (HuR). Cancer Lett. 421, 145–151. 10.1016/j.canlet.2018.02.026 29471005

[B6] Ale-Agha N. Galban S. Sobieroy C. Abdelmohsen K. Gorospe M. Sies H. (2009). HuR Regulates gap Junctional Intercellular Communication by Controlling β-catenin Levels and Adherens junction Integrity. Hepatology 50, 1567–1576. 10.1002/hep.23146 19676129 PMC2784158

[B7] Aranda-Abreu G. E. Behar L. Chung S. Furneaux H. Ginzburg I. (1999). Embryonic Lethal Abnormal Vision-like RNA-Binding Proteins Regulate Neurite Outgrowth and Tau Expression in PC12 Cells. J. Neurosci. 19, 6907–6917. 10.1523/jneurosci.19-16-06907.1999 10436048 PMC6782881

[B8] Bakheet T. Williams B. R. Khabar K. S. (2006). ARED 3.0: the Large and Diverse AU-Rich Transcriptome. Nucleic Acids Res. 34, D111–D114. 10.1093/nar/gkj052 16381826 PMC1347415

[B9] Barbisan F. Mazzucchelli R. Santinelli A. Lopez-Beltran A. Cheng L. Scarpelli M. (2009). Overexpression of ELAV-like Protein HuR Is Associated with Increased COX-2 Expression in Atrophy, High-Grade Prostatic Intraepithelial Neoplasia, and Incidental Prostate Cancer in Cystoprostatectomies. Eur. Urol. 56, 105–112. 10.1016/j.eururo.2008.04.043 18468781

[B10] Barreau C. Paillard L. Osborne H. B. (2005). AU-rich Elements and Associated Factors: Are There Unifying Principles? Nucleic Acids Res. 33, 7138–7150. 10.1093/nar/gki1012 16391004 PMC1325018

[B11] Beauchamp P. Nassif C. Hillock S. van der Giessen K. von Roretz C. Jasmin B. J. (2010). The Cleavage of HuR Interferes with its Transportin-2-Mediated Nuclear Import and Promotes Muscle Fiber Formation. Cell Death Differ 17, 1588–1599. 10.1038/cdd.2010.34 20379198

[B12] Benyahia B. Liblau R. Merle-Béral H. l. n. Tourani J.-M. Dalmau J. Delattre J.-Y. (1999). Cell-mediated Autoimmunity in Paraneoplastic Neurological Syndromes with Anti-hu Antibodies. Ann. Neurol. 45, 162–167. 10.1002/1531-8249(199902)45:2<162::aid-ana5>3.0.co;2-r 9989617

[B13] Bibli S.-I. Hu J. Sigala F. Wittig I. Heidler J. Zukunft S. (2019). Cystathionine γ Lyase Sulfhydrates the RNA Binding Protein Human Antigen R to Preserve Endothelial Cell Function and Delay Atherogenesis. Circulation 139, 101–114. 10.1161/circulationaha.118.034757 29970364

[B14] Blanco F. F. Jimbo M. Wulfkuhle J. Gallagher I. Deng J. Enyenihi L. (2016). The mRNA-Binding Protein HuR Promotes Hypoxia-Induced Chemoresistance through Posttranscriptional Regulation of the Proto-Oncogene PIM1 in Pancreatic Cancer Cells. Oncogene 35, 2529–2541. 10.1038/onc.2015.325 26387536 PMC6818728

[B15] Bronicki L. M. Belanger G. Jasmin B. J. (2012). Characterization of Multiple Exon 1 Variants in Mammalian HuD mRNA and Neuron-specific Transcriptional Control via Neurogenin 2. J. Neurosci. 32, 11164–11175. 10.1523/jneurosci.2247-12.2012 22895702 PMC6621197

[B16] Bussel J. Kulasekararaj A. Cooper N. Verma A. Steidl U. Semple J. W. (2019). Mechanisms and Therapeutic Prospects of Thrombopoietin Receptor Agonists. Semin. Hematol. 56, 262–278. 10.1053/j.seminhematol.2019.09.001 31836033

[B17] Cai J. Wang H. Jiao X. Huang R. Qin Q. Zhang J. (2019). The RNA-Binding Protein HuR Confers Oxaliplatin Resistance of Colorectal Cancer by Upregulating CDC6. Mol. Cancer Ther. 18, 1243–1254. 10.1158/1535-7163.Mct-18-0945 31064870

[B18] Campos A. R. And D. G. White K. (1985). Mutant Alleles at the Locus Elav in *Drosophila melanogaster* lead to Nervous System Defects. A Developmental-Genetic Analysis. J. Neurogenet. 2, 197–218. 10.3109/01677068509100150 3926976

[B19] Cao C. Sun J. Zhang D. Guo X. Xie L. Li X. (2015). The Long Intergenic Noncoding RNA UFC1, a Target of MicroRNA 34a, Interacts with the mRNA Stabilizing Protein HuR to Increase Levels of β-Catenin in HCC Cells. Gastroenterology 148, 415–426. 10.1053/j.gastro.2014.10.012 25449213

[B20] Casolaro V. Fang X. Tancowny B. Fan J. Wu F. Srikantan S. (2008). Posttranscriptional Regulation of IL-13 in T Cells: Role of the RNA-Binding Protein HuR. J. Allergy Clin. Immunol. 121, 853–859. e854. 10.1016/j.jaci.2007.12.1166 18279945 PMC2666917

[B21] Chae M.-J. Sung H. Y. Kim E.-H. Lee M. Kwak H. Chae C. H. (2009). Chemical Inhibitors Destabilize HuR Binding to the AU-Rich Element of TNF-α mRNA. Exp. Mol. Med. 41, 824–831. 10.3858/emm.2009.41.11.088 19949288 PMC2788736

[B22] Chand S. N. Zarei M. Schiewer M. J. Kamath A. R. Romeo C. Lal S. (2017). Posttranscriptional Regulation of PARG mRNA by HuR Facilitates DNA Repair and Resistance to PARP Inhibitors. Cancer Res. 77, 5011–5025. 10.1158/0008-5472.Can-16-2704 28687616 PMC5663502

[B23] Chen C. Y. Xu N. Shyu A. B. (1995). mRNA Decay Mediated by Two Distinct AU-Rich Elements from C-Fos and Granulocyte-Macrophage colony-stimulating Factor Transcripts: Different Deadenylation Kinetics and Uncoupling from Translation. Mol. Cell Biol 15, 5777–5788. 10.1128/mcb.15.10.5777 7565731 PMC230830

[B24] Chen J. Wu Y. Luo X. Jin D. Zhou W. Ju Z. (2021). Circular RNA circRHOBTB3 Represses Metastasis by Regulating the HuR-Mediated mRNA Stability of PTBP1 in Colorectal Cancer. Theranostics 11, 7507–7526. 10.7150/thno.59546 34158864 PMC8210600

[B25] Chen R. Lei S. She Y. Zhou S. Shi H. Li C. (2021). Lnc-GD2H Promotes Proliferation by Forming a Feedback Loop with C-Myc and Enhances Differentiation through Interacting with NACA to Upregulate Myog in C2C12 Myoblasts. Front. Cell Dev. Biol. 9, 671857. 10.3389/fcell.2021.671857 34490239 PMC8416608

[B26] Chen Y. Yang F. Fang E. Xiao W. Mei H. Li H. (2019). Circular RNA circAGO2 Drives Cancer Progression through Facilitating HuR-Repressed Functions of AGO2-miRNA Complexes. Cell Death Differ 26, 1346–1364. 10.1038/s41418-018-0220-6 30341421 PMC6748083

[B27] Cheng X. Gu X. Xia T. Ma Z. Yang Z. Feng H. L. (2021). HuB and HuD Repress Telomerase Activity by Dissociating HuR from TERC. Nucleic Acids Res. 49, 2848–2858. 10.1093/nar/gkab062 33589924 PMC7969021

[B28] Colombrita C. Silani V. Ratti A. (2013). ELAV Proteins along Evolution: Back to the Nucleus? Mol. Cell Neurosci. 56, 447–455. 10.1016/j.mcn.2013.02.003 23439364

[B29] Costantino C. L. Witkiewicz A. K. Kuwano Y. Cozzitorto J. A. Kennedy E. P. Dasgupta A. (2009). The Role of HuR in Gemcitabine Efficacy in Pancreatic Cancer: HuR Up-Regulates the Expression of the Gemcitabine Metabolizing Enzyme Deoxycytidine Kinase. Cancer Res. 69, 4567–4572. 10.1158/0008-5472.Can-09-0371 19487279 PMC2744447

[B30] Cui Y. H. Feng Q. Y. Liu Q. Li H. Y. Song X. L. Hu Z. X. (2020). Posttranscriptional Regulation of MMP‐9 by HuR Contributes to IL‐1β‐induced Pterygium Fibroblast Migration and Invasion. J. Cell Physiol 235, 5130–5140. 10.1002/jcp.29387 31691974

[B31] D'Alessandro V. Muscarella L. A. Copetti M. Zelante L. Carella M. Vendemiale G. (2008). Molecular Detection of Neuron-specific ELAV-Like-Positive Cells in the Peripheral Blood of Patients with Small-Cell Lung Cancer. Cell Oncol 30, 291–297. 10.3233/clo-2008-0424 18607064 PMC4618425

[B32] De Santis R. Santini L. Colantoni A. Peruzzi G. de Turris V. Alfano V. (2017). FUS Mutant Human Motoneurons Display Altered Transcriptome and microRNA Pathways with Implications for ALS Pathogenesis. Stem Cell Rep. 9, 1450–1462. 10.1016/j.stemcr.2017.09.004 PMC5830977 28988989

[B33] de Silanes I. L. Fan J. Yang X. Zonderman A. B. Potapova O. Pizer E. S. (2003). Role of the RNA-Binding Protein HuR in colon Carcinogenesis. Oncogene 22, 7146–7154. 10.1038/sj.onc.1206862 14562043

[B34] DeBoer E. M. Azevedo R. Vega T. A. Brodkin J. Akamatsu W. Okano H. (2014). Prenatal Deletion of the RNA-Binding Protein HuD Disrupts Postnatal Cortical Circuit Maturation and Behavior. J. Neurosci. 34, 3674–3686. 10.1523/jneurosci.3703-13.2014 24599466 PMC3942583

[B35] Denkert C. Koch I. von Keyserlingk N. Noske A. Niesporek S. Dietel M. (2006). Expression of the ELAV-like Protein HuR in Human colon Cancer: Association with Tumor Stage and Cyclooxygenase-2. Mod. Pathol. 19, 1261–1269. 10.1038/modpathol.3800645 16799479

[B36] Denkert C. Weichert W. Pest S. Koch I. Licht D. Köbel M. (2004). Overexpression of the Embryonic-Lethal Abnormal Vision-like Protein HuR in Ovarian Carcinoma Is a Prognostic Factor and Is Associated with Increased Cyclooxygenase 2 Expression. Cancer Res. 64, 189–195. 10.1158/0008-5472.can-03-1987 14729623

[B37] Deschenes-Furry J. Mousavi K. Bolognani F. Neve R. L. Parks R. J. Perrone-Bizzozero N. I. (2007). The RNA-Binding Protein HuD Binds Acetylcholinesterase mRNA in Neurons and Regulates its Expression after Axotomy. J. Neurosci. 27, 665–675. 10.1523/jneurosci.4626-06.2007 17234598 PMC6672799

[B38] Dixon D. A. Tolley N. D. King P. H. Nabors L. B. McIntyre T. M. Zimmerman G. A. (2001). Altered Expression of the mRNA Stability Factor HuR Promotes Cyclooxygenase-2 Expression in colon Cancer Cells. J. Clin. Invest. 108, 1657–1665. 10.1172/jci12973 11733561 PMC200983

[B39] Dou Q. Xu Y. Zhu Y. Hu Y. Yan Y. Yan H. (2019). LncRNA FAM83H-AS1 Contributes to the Radioresistance, Proliferation, and Metastasis in Ovarian Cancer through Stabilizing HuR Protein. Eur. J. Pharmacol. 852, 134–141. 10.1016/j.ejphar.2019.03.002 30831080

[B40] Durie D. Lewis S. M. Liwak U. Kisilewicz M. Gorospe M. Holcik M. (2011). RNA-binding Protein HuR Mediates Cytoprotection through Stimulation of XIAP Translation. Oncogene 30, 1460–1469. 10.1038/onc.2010.527 21102524 PMC3514411

[B41] Ehrlich D. Wang B. Lu W. Dowling P. Yuan R. (2014). Intratumoral Anti-HuD Immunotoxin Therapy for Small Cell Lung Cancer and Neuroblastoma. J. Hematol. Oncol. 7, 91. 10.1186/s13045-014-0091-3 25523825 PMC4293823

[B42] Erkinheimo T. L. Lassus H. Sivula A. Sengupta S. Furneaux H. Hla T. (2003). Cytoplasmic HuR Expression Correlates with Poor Outcome and with Cyclooxygenase 2 Expression in Serous Ovarian Carcinoma. Cancer Res. 63, 7591–7594. http://aacrjournals.org/cancerres/article-pdf/63/22/7591/2509724/zch02203007591.pdf. 14633672

[B43] Faheem S. A. El- Sayed N. M. Moustafa Y. M. Saeed N. M. Hazem R. M. (2022). Pyrvinium Pamoate Ameliorates Cyclosporin A- Induced Hepatotoxicity via the Modulation of Wnt/β-Catenin Signaling and Upregulation of PPAR-γ. Int. Immunopharmacology 104, 108538. 10.1016/j.intimp.2022.108538 35074592

[B44] Fan X. C. Myer V. E. Steitz J. A. (1997). AU-rich Elements Target Small Nuclear RNAs as Well as mRNAs for Rapid Degradation. Genes Dev. 11, 2557–2568. 10.1101/gad.11.19.2557 9334320 PMC316563

[B45] Fan X. C. Steitz J. A. (1998). Overexpression of HuR, a Nuclear-Cytoplasmic Shuttling Protein, Increases the Invivo Stability of ARE-Containing mRNAs. Embo j 17, 3448–3460. 10.1093/emboj/17.12.3448 9628880 PMC1170681

[B46] Fan X. C. Steitz J. A. (1998). HNS, a Nuclear-Cytoplasmic Shuttling Sequence in HuR. Proc. Natl. Acad. Sci. 95, 15293–15298. 10.1073/pnas.95.26.15293 9860962 PMC28036

[B47] Farooq F. Balabanian S. Liu X. Holcik M. MacKenzie A. (2009). p38 Mitogen-Activated Protein Kinase Stabilizes SMN mRNA through RNA Binding Protein HuR. Hum. Mol. Genet. 18, 4035–4045. 10.1093/hmg/ddp352 19648294

[B48] Filippova N. Yang X. Ananthan S. Calano J. Pathak V. Bratton L. (2021). Targeting the HuR Oncogenic Role with a New Class of Cytoplasmic Dimerization Inhibitors. Cancer Res. 81, 2220–2233. 10.1158/0008-5472.Can-20-2858 33602784 PMC8137579

[B49] Fukao A. Sasano Y. Imataka H. Inoue K. Sakamoto H. Sonenberg N. (2009). The ELAV Protein HuD Stimulates Cap-dependent Translation in a Poly(A)- and eIF4A-dependent Manner. Mol. Cell 36, 1007–1017. 10.1016/j.molcel.2009.11.013 20064466

[B50] Gherzi R. Lee K.-Y. Briata P. Wegmüller D. Moroni C. Karin M. (2004). A KH Domain RNA Binding Protein, KSRP, Promotes ARE-Directed mRNA Turnover by Recruiting the Degradation Machinery. Mol. Cell 14, 571–583. 10.1016/j.molcel.2004.05.002 15175153

[B51] Ghosh M. Aguila H. L. Michaud J. Ai Y. Wu M.-T. Hemmes A. (2009). Essential Role of the RNA-Binding Protein HuR in Progenitor Cell Survival in Mice. J. Clin. Invest. 119, 3530–3543. 10.1172/jci38263 19884656 PMC2786787

[B52] Good P. J. (1995). A Conserved Family of Elav-like Genes in Vertebrates. Proc. Natl. Acad. Sci. 92, 4557–4561. 10.1073/pnas.92.10.4557 7753842 PMC41983

[B53] Gouble A. Morello D. (2000). Synchronous and Regulated Expression of Two AU-Binding Proteins, AUF1 and HuR, throughout Murine Development. Oncogene 19, 5377–5384. 10.1038/sj.onc.1203910 11103939

[B54] Gratacós F. M. Brewer G. (2010). The Role of AUF1 in Regulated mRNA Decay. Wiley Interdiscip. Rev. RNA 1, 457–473. 10.1002/wrna.26 21956942 PMC3608466

[B55] Guo J. Lv J. Chang S. Chen Z. Lu W. Xu C. (2016). Inhibiting Cytoplasmic Accumulation of HuR Synergizes Genotoxic Agents in Urothelial Carcinoma of the Bladder. Oncotarget 7, 45249–45262. 10.18632/oncotarget.9932 27303922 PMC5216720

[B56] Guo X. Hartley R. S. (2006). HuR Contributes to Cyclin E1 Deregulation in MCF-7 Breast Cancer Cells. Cancer Res. 66, 7948–7956. 10.1158/0008-5472.Can-05-4362 16912169

[B57] Guo X. Wu Y. Hartley R. (2009). MicroRNA-125a Represses Cell Growth by Targeting HuR in Breast Cancer. RNA Biol. 6, 575–583. 10.4161/rna.6.5.10079 19875930 PMC3645467

[B58] Hambardzumyan D. Sergent-Tanguy S. Thinard R. Bonnamain V. Masip M. Fabre A. (2009). AUF1 and Hu Proteins in the Developing Rat Brain: Implication in the Proliferation and Differentiation of Neural Progenitors. J. Neurosci. Res. 87, 1296–1309. 10.1002/jnr.21957 19115409

[B59] Hao le T. Duy P. Q. An M. Talbot J. Iyer C. C. Wolman M. (2017). HuD and the Survival Motor Neuron Protein Interact in Motoneurons and Are Essential for Motoneuron Development, Function, and mRNA Regulation. J. Neurosci. 37, 11559–11571. 10.1523/jneurosci.1528-17.2017 29061699 PMC5707763

[B60] Hatanaka T. Higashino F. Tei K. Yasuda M. (2019). The Neural ELAVL Protein HuB Enhances Endogenous Proto-Oncogene Activation. Biochem. Biophysical Res. Commun. 517, 330–337. 10.1016/j.bbrc.2019.07.089 31358321

[B61] He X. B. Wu Y. Huang H. Guo F. (2022). A Novel Histone Deacetylase Inhibitor‐based Approach to Eliminate Microglia and Retain Astrocyte Properties in Glial Cell Culture. J. Neurochem. 10.1111/jnc.15581 35092690

[B62] Heinonen M. Fagerholm R. Aaltonen K. Kilpivaara O. Aittomäki K. Blomqvist C. (2007). Prognostic Role of HuR in Hereditary Breast Cancer. Clin. Cancer Res. 13, 6959–6963. 10.1158/1078-0432.Ccr-07-1432 18056170

[B63] Heinonen M. Hemmes A. Salmenkivi K. Abdelmohsen K. Vilén S.-T. Laakso M. (2011). Role of RNA Binding Protein HuR in Ductal Carcinoma *In Situ* of the Breast. J. Pathol. 224, 529–539. 10.1002/path.2889 21480233 PMC3504799

[B64] Hostetter C. Licata L. A. Costantino C. L. Witkiewicz A. Yeo C. Brody J. R. (2008). Cytoplasmic Accumulation of the RNA Binding Protein HuR Is central to Tamoxifen Resistance in Estrogen Receptor Positive Breast Cancer Cells. Cancer Biol. Ther. 7, 1496–1506. 10.4161/cbt.7.9.6490 18769129

[B65] Hufnagel H. Hakim P. Lima A. Hollfelder F. (2009). Fluid Phase Endocytosis Contributes to Transfection of DNA by PEI-25. Mol. Ther. 17, 1411–1417. 10.1038/mt.2009.121 19532143 PMC2835228

[B66] Ishihara Y. Tsuno S. Kuwamoto S. Yamashita T. Endo Y. Hasegawa J. (2014). Hsa-miR-520d Converts Fibroblasts into CD105+ Populations. Drugs R. D 14, 253–264. 10.1007/s40268-014-0064-6 25303886 PMC4269822

[B67] Kakuguchi W. Nomura T. Kitamura T. Otsuguro S. Matsushita K. Sakaitani M. (2018). Suramin, Screened from an Approved Drug Library, Inhibits HuR Functions and Attenuates Malignant Phenotype of Oral Cancer Cells. Cancer Med. 7, 6269–6280. 10.1002/cam4.1877 30449075 PMC6308099

[B68] Kang M. J. Ryu B. K. Lee M. G. Han J. Lee J. H. Ha T. K. (2008). NF-κB Activates Transcription of the RNA-Binding Factor HuR, via PI3K-AKT Signaling, to Promote Gastric Tumorigenesis. Gastroenterology 135, 2030–2042. 10.1053/j.gastro.2008.08.009 18824170

[B69] Kaur K. Wu X. Fields J. K. Johnson D. K. Lan L. Pratt M. (2017). The Fungal Natural Product Azaphilone-9 Binds to HuR and Inhibits HuR-RNA Interaction *In Vitro* . PLoS One 12, e0175471. 10.1371/journal.pone.0175471 28414767 PMC5393604

[B70] Ke Y. Han Y. Guo X. Wen J. Wang K. Jiang X. (2017). PARP1 Promotes Gene Expression at the post-transcriptional Level by Modulating the RNA-Binding Protein HuR. Nat. Commun. 8, 14632. 10.1038/ncomms14632 28272405 PMC5344980

[B71] Ke Y. Lv X. Fu X. Zhang J. Bohio A. A. Zeng X. (2021). Poly(ADP-ribosyl)ation Enhances HuR Oligomerization and Contributes to Pro-inflammatory Gene mRNA Stabilization. Cell. Mol. Life Sci. 78, 1817–1835. 10.1007/s00018-020-03618-4 32789690 PMC7904744

[B72] Kim C. Jeong D. E. Heo S. Ji E. Rho J. G. Jung M. (2018). Reduced Expression of the RNA-Binding Protein HuD in Pancreatic Neuroendocrine Tumors Correlates with Low p27Kip1 Levels and Poor Prognosis. J. Pathol. 246, 231–243. 10.1002/path.5135 30014466 PMC6150781

[B73] Kim H. H. Abdelmohsen K. Lal A. Pullmann R. Jr. Yang X. Galban S. (2008). Nuclear HuR Accumulation through Phosphorylation by Cdk1. Genes Dev. 22, 1804–1815. 10.1101/gad.1645808 18593881 PMC2492667

[B74] King P. H. (1997). Differential Expression of the Neuroendocrine genesHel-N1 and HuD in Small-Cell Lung Carcinoma: Evidence for Down-Regulation of HuD in the Variant Phenotype. Int. J. Cancer 74, 378–382. 10.1002/(sici)1097-0215(19970822)74:4<378::aid-ijc3>3.0.co;2-s 9291425

[B75] Kraushar M. L. Thompson K. Wijeratne H. R. S. Viljetic B. Sakers K. Marson J. W. (2014). Temporally Defined Neocortical Translation and Polysome Assembly Are Determined by the RNA-Binding Protein Hu Antigen R. Proc. Natl. Acad. Sci. U.S.A. 111, E3815–E3824. 10.1073/pnas.1408305111 25157170 PMC4246959

[B76] Krishnamurthy P. Rajasingh J. Lambers E. Qin G. Losordo D. W. Kishore R. (2009). IL-10 Inhibits Inflammation and Attenuates Left Ventricular Remodeling after Myocardial Infarction via Activation of STAT3 and Suppression of HuR. Circ. Res. 104, e9–18. 10.1161/circresaha.108.188243 19096025 PMC2774810

[B77] Lal P. Cerofolini L. D’Agostino V. G. Zucal C. Fuccio C. Bonomo I. (2017). Regulation of HuR Structure and Function by Dihydrotanshinone-I. Nucleic Acids Res. 45, 9514–9527. 10.1093/nar/gkx623 28934484 PMC5766160

[B78] Lan Y. Xiao X. He Z. Luo Y. Wu C. Li L. (2018). Long Noncoding RNA OCC-1 Suppresses Cell Growth through Destabilizing HuR Protein in Colorectal Cancer. Nucleic Acids Res. 46, 5809–5821. 10.1093/nar/gky214 29931370 PMC6009600

[B79] Latorre E. Tebaldi T. Viero G. Spartà A. M. Quattrone A. Provenzani A. (2012). Downregulation of HuR as a New Mechanism of Doxorubicin Resistance in Breast Cancer Cells. Mol. Cancer 11, 13. 10.1186/1476-4598-11-13 22436134 PMC3325864

[B80] Lazarova D. L. Spengler B. A. Biedler J. L. Ross R. A. (1999). HuD, a Neuronal-specific RNA-Binding Protein, Is a Putative Regulator of N-Myc Pre-mRNA Processing/stability in Malignant Human Neuroblasts. Oncogene 18, 2703–2710. 10.1038/sj.onc.1202621 10348344

[B81] Leandersson K. Riesbeck K. Andersson T. (2006). Wnt-5a mRNA Translation Is Suppressed by the Elav-like Protein HuR in Human Breast Epithelial Cells. Nucleic Acids Res. 34, 3988–3999. 10.1093/nar/gkl571 16914445 PMC1557823

[B82] Lee H. K. Jeong S. (2006). β-Catenin Stabilizes Cyclooxygenase-2 mRNA by Interacting with AU-Rich Elements of 3′-UTR. Nucleic Acids Res. 34, 5705–5714. 10.1093/nar/gkl698 17040897 PMC1636482

[B83] Lee J. H. Jung M. Hong J. Kim M. K. Chung I. K. (2018). Loss of RNA-Binding Protein HuR Facilitates Cellular Senescence through Posttranscriptional Regulation of TIN2 mRNA. Nucleic Acids Res. 46, 4271–4285. 10.1093/nar/gky223 29584879 PMC5934620

[B84] Legnini I. Morlando M. Mangiavacchi A. Fatica A. Bozzoni I. (2014). A Feedforward Regulatory Loop between HuR and the Long Noncoding RNA linc-MD1 Controls Early Phases of Myogenesis. Mol. Cell 53, 506–514. 10.1016/j.molcel.2013.12.012 24440503 PMC3919156

[B85] Li X.-X. Xiao L. Chung H. K. Ma X.-X. Liu X. Song J.-L. (2020). Interaction between HuR and circPABPN1 Modulates Autophagy in the Intestinal Epithelium by Altering ATG16L1 Translation. Mol. Cell Biol 40 (6), e00492-19. 10.1128/mcb.00492-19 31932481 PMC7048268

[B86] Li Y. Zheng Q. Bao C. Li S. Guo W. Zhao J. (2015). Circular RNA Is Enriched and Stable in Exosomes: a Promising Biomarker for Cancer Diagnosis. Cell Res 25, 981–984. 10.1038/cr.2015.82 26138677 PMC4528056

[B87] Liu B. Yang G. Wang X. Liu J. Lu Z. Wang Q. (2020). CircBACH1 (Hsa_circ_0061395) Promotes Hepatocellular Carcinoma Growth by Regulating P27 Repression via HuR. J. Cell Physiol 235, 6929–6941. 10.1002/jcp.29589 32003018

[B88] Liu H. Lan T. Li H. Xu L. Chen X. Liao H. (2021). Circular RNA circDLC1 Inhibits MMP1-Mediated Liver Cancer Progression via Interaction with HuR. Theranostics 11, 1396–1411. 10.7150/thno.53227 33391541 PMC7738888

[B89] Liu L. Xiao L. Chung H. K. Kwon M. S. Li X.-X. Wu N. (2019). RNA-binding Protein HuR Regulates Rac1 Nucleocytoplasmic Shuttling through Nucleophosmin in the Intestinal Epithelium. Cell Mol. Gastroenterol. Hepatol. 8, 475–486. 10.1016/j.jcmgh.2019.06.002 31195150 PMC6718926

[B90] Liu S. Jiang X. Lu H. Xing M. Qiao Y. Zhang C. (2020). HuR (Human Antigen R) Regulates the Contraction of Vascular Smooth Muscle and Maintains Blood Pressure. Atvb 40, 943–957. 10.1161/atvbaha.119.313897 32075416

[B91] Loffreda A. Nizzardo M. Arosio A. Ruepp M.-D. Calogero R. A. Volinia S. (2020). miR-129-5p: A Key Factor and Therapeutic Target in Amyotrophic Lateral Sclerosis. Prog. Neurobiol. 190, 101803. 10.1016/j.pneurobio.2020.101803 32335272

[B92] Lu Y.-C. Chang S.-H. Hafner M. Li X. Tuschl T. Elemento O. (2014). ELAVL1 Modulates Transcriptome-wide miRNA Binding in Murine Macrophages. Cell Rep. 9, 2330–2343. 10.1016/j.celrep.2014.11.030 25533351 PMC4277505

[B93] Lucchesi C. Sheikh M. S. Huang Y. (2016). Negative Regulation of RNA-Binding Protein HuR by Tumor-Suppressor ECRG2. Oncogene 35, 2565–2573. 10.1038/onc.2015.339 26434587

[B94] Lykke-Andersen J. Wagner E. (2005). Recruitment and Activation of mRNA Decay Enzymes by Two ARE-Mediated Decay Activation Domains in the Proteins TTP and BRF-1. Genes Dev. 19, 351–361. 10.1101/gad.1282305 15687258 PMC546513

[B95] Marchesi N. Thongon N. Pascale A. Provenzani A. Koskela A. Korhonen E. (2018). Autophagy Stimulus Promotes Early HuR Protein Activation and p62/SQSTM1 Protein Synthesis in ARPE-19 Cells by Triggering Erk1/2, p38MAPK, and JNK Kinase Pathways. Oxidative Med. Cell. longevity 2018, 1–15. 10.1155/2018/4956080 PMC5822911 29576851

[B96] Masuda K. Abdelmohsen K. Kim M. M. Srikantan S. Lee E. K. Tominaga K. (2011). Global Dissociation of HuR-mRNA Complexes Promotes Cell Survival after Ionizing Radiation. Embo j 30, 1040–1053. 10.1038/emboj.2011.24 21317874 PMC3061031

[B97] Matsye P. Zheng L. Si Y. Kim S. Luo W. Crossman D. K. (2017). HuR Promotes the Molecular Signature and Phenotype of Activated Microglia: Implications for Amyotrophic Lateral Sclerosis and Other Neurodegenerative Diseases. Glia 65, 945–963. 10.1002/glia.23137 28300326 PMC7944581

[B98] Mazan-Mamczarz K. Galbán S. de Silanes I. L. Martindale J. L. Atasoy U. Keene J. D. (2003). RNA-binding Protein HuR Enhances P53 Translation in Response to Ultraviolet Light Irradiation. Proc. Natl. Acad. Sci. U.S.A. 100, 8354–8359. 10.1073/pnas.1432104100 12821781 PMC166233

[B99] Mazan-Mamczarz K. Hagner P. R. Corl S. Srikantan S. Wood W. H. Becker K. G. (2008). Post-transcriptional Gene Regulation by HuR Promotes a More Tumorigenic Phenotype. Oncogene 27, 6151–6163. 10.1038/onc.2008.215 18641687 PMC2575016

[B100] Mazroui R. Di Marco S. Clair E. von Roretz C. Tenenbaum S. A. Keene J. D. (2008). Caspase-mediated Cleavage of HuR in the Cytoplasm Contributes to Pp32/PHAP-I Regulation of Apoptosis. J. Cell Biol 180, 113–127. 10.1083/jcb.200709030 18180367 PMC2213623

[B101] Meisner N.-C. Hintersteiner M. Mueller K. Bauer R. Seifert J.-M. Naegeli H.-U. (2007). Identification and Mechanistic Characterization of Low-Molecular-Weight Inhibitors for HuR. Nat. Chem. Biol. 3, 508–515. 10.1038/nchembio.2007.14 17632515

[B102] Meng Z. King P. H. Nabors L. B. Jackson N. L. Chen C. Y. Emanuel P. D. (2005). The ELAV RNA-Stability Factor HuR Binds the 5'-untranslated Region of the Human IGF-IR Transcript and Differentially Represses Cap-dependent and IRES-Mediated Translation. Nucleic Acids Res. 33, 2962–2979. 10.1093/nar/gki603 15914670 PMC1140080

[B103] Mitsunari K. Miyata Y. Asai A. Matsuo T. Shida Y. Hakariya T. (2016). Human Antigen R Is Positively Associated with Malignant Aggressiveness via Upregulation of Cell Proliferation, Migration, and Vascular Endothelial Growth Factors and Cyclooxygenase-2 in Prostate Cancer. Translational Res. 175, 116–128. 10.1016/j.trsl.2016.04.002 27140699

[B104] Mukherjee J. Ohba S. See W. L. Phillips J. J. Molinaro A. M. Pieper R. O. (2016). PKM2 Uses Control of HuR Localization to Regulate P27 and Cell Cycle Progression in Human Glioblastoma Cells. Int. J. Cancer 139, 99–111. 10.1002/ijc.30041 26874904 PMC6615049

[B105] Muralidharan R. Mehta M. Ahmed R. Roy S. Xu L. Aubé J. (2017). HuR-targeted Small Molecule Inhibitor Exhibits Cytotoxicity towards Human Lung Cancer Cells. Sci. Rep. 7, 9694. 10.1038/s41598-017-07787-4 28855578 PMC5577245

[B106] Myer V. E. Fan X. C. Steitz J. A. (1997). Identification of HuR as a Protein Implicated in AUUUA-Mediated mRNA Decay. Embo j 16, 2130–2139. 10.1093/emboj/16.8.2130 9155038 PMC1169815

[B107] Nabors L. B. Gillespie G. Y. Harkins L. King P. H. (2001). HuR, a RNA Stability Factor, Is Expressed in Malignant Brain Tumors and Binds to Adenine- and Uridine-Rich Elements within the 3' Untranslated Regions of Cytokine and Angiogenic Factor mRNAs. Cancer Res. 61, 2154–2161. http://aacrjournals.org/cancerres/article-pdf/61/5/2154/2493096/ch050102154.pdf. 11280780

[B108] Ohwada A. Nagaoka I. Takahashi F. Tominaga S. Fukuchi Y. (1999). DNA Vaccination against HuD Antigen Elicits Antitumor Activity in a Small-Cell Lung Cancer Murine Model. Am. J. Respir. Cell Mol Biol 21, 37–43. 10.1165/ajrcmb.21.1.3625 10385591

[B109] Peng W.-X. Koirala P. Zhang W. Ni C. Wang Z. Yang L. (2020). lncRNA RMST Enhances DNMT3 Expression through Interaction with HuR. Mol. Ther. 28, 9–18. 10.1016/j.ymthe.2019.09.024 31636039 PMC6953777

[B110] Quattrone A. Pascale A. Nogues X. Zhao W. Gusev P. Pacini A. (2001). Posttranscriptional Regulation of Gene Expression in Learning by the Neuronal ELAV-like mRNA-Stabilizing Proteins. Proc. Natl. Acad. Sci. 98, 11668–11673. 10.1073/pnas.191388398 11573004 PMC58787

[B111] Raspaglio G. De Maria I. Filippetti F. Martinelli E. Zannoni G. F. Prislei S. (2010). HuR Regulates β-Tubulin Isotype Expression in Ovarian Cancer. Cancer Res. 70, 5891–5900. 10.1158/0008-5472.Can-09-4656 20587520

[B112] Ripin N. Boudet J. Duszczyk M. M. Hinniger A. Faller M. Krepl M. (2019). Molecular Basis for AU-Rich Element Recognition and Dimerization by the HuR C-Terminal RRM. Proc. Natl. Acad. Sci. USA 116, 2935–2944. 10.1073/pnas.1808696116 30718402 PMC6386705

[B113] Rogulja-Ortmann A. Picao-Osorio J. Villava C. Patraquim P. Lafuente E. Aspden J. (2014). The RNA-Binding Protein ELAV Regulates Hox RNA Processing, Expression and Function within the Drosophila Nervous System. Development 141, 2046–2056. 10.1242/dev.101519 24803653 PMC4132933

[B114] Schultz C. W. Preet R. Dhir T. Dixon D. A. Brody J. R. (2020). Understanding and Targeting the Disease‐related RNA Binding Protein Human Antigen R (HuR). Wiley Interdiscip. Rev. RNA 11, e1581. 10.1002/wrna.1581 31970930 PMC7482136

[B115] Shen L. Han J. Wang H. Meng Q. Chen L. Liu Y. (2019). Cachexia-related Long Noncoding RNA, CAAlnc1, Suppresses Adipogenesis by Blocking the Binding of HuR to Adipogenic Transcription Factor mRNAs. Int. J. Cancer 145, 1809–1821. 10.1002/ijc.32236 30807648

[B116] Siang D. T. C. Lim Y. C. Kyaw A. M. M. Win K. N. Chia S. Y. Degirmenci U. (2020). The RNA-Binding Protein HuR Is a Negative Regulator in Adipogenesis. Nat. Commun. 11, 213. 10.1038/s41467-019-14001-8 31924774 PMC6954112

[B117] Simion V. Zhou H. Haemmig S. Pierce J. B. Mendes S. Tesmenitsky Y. (2020). A Macrophage-specific lncRNA Regulates Apoptosis and Atherosclerosis by Tethering HuR in the Nucleus. Nat. Commun. 11, 6135. 10.1038/s41467-020-19664-2 33262333 PMC7708640

[B118] Skliris A. Papadaki O. Kafasla P. Karakasiliotis I. Hazapis O. Reczko M. (2015). Neuroprotection Requires the Functions of the RNA-Binding Protein HuR. Cell Death Differ 22, 703–718. 10.1038/cdd.2014.158 25301069 PMC4392069

[B119] Song J. VanBuskirk J. A. Merbs S. L. (2022). Regulation of Opsin Gene Expression by DNA Methylation and Histone Acetylation. Ijms 23, 1408. 10.3390/ijms23031408 35163334 PMC8836077

[B120] Sun C. Han B. Zhai Y. Zhao H. Li X. Qian J. (2022). Dihydrotanshinone I Inhibits Ovarian Tumor Growth by Activating Oxidative Stress through Keap1-Mediated Nrf2 Ubiquitination Degradation. Free Radic. Biol. Med. 180, 220–235. 10.1016/j.freeradbiomed.2022.01.015 35074488

[B121] Suswam E. A. Nabors L. B. Huang Y. Yang X. King P. H. (2005). IL-1? Induces Stabilization of IL-8 mRNA in Malignant Breast Cancer Cellsvia the 3? Untranslated Region: Involvement of Divergent RNA-Binding Factors HuR, KSRP and TIAR. Int. J. Cancer 113, 911–919. 10.1002/ijc.20675 15514971

[B122] Toba G. White K. (2008). The Third RNA Recognition Motif of Drosophila ELAV Protein Has a Role in Multimerization. Nucleic Acids Res. 36, 1390–1399. 10.1093/nar/gkm1168 18203745 PMC2275111

[B123] Wang F. Tidei J. J. Polich E. D. Gao Y. Zhao H. Perrone-Bizzozero N. I. (2015). Positive Feedback between RNA-Binding Protein HuD and Transcription Factor SATB1 Promotes Neurogenesis. Proc. Natl. Acad. Sci. U.S.A. 112, E4995–E5004. 10.1073/pnas.1513780112 26305964 PMC4568658

[B124] Wang J. Leavenworth J. W. Hjelmeland A. B. Smith R. Patel N. Borg B. (2019). Deletion of the RNA Regulator HuR in Tumor‐associated Microglia and Macrophages Stimulates Anti‐tumor Immunity and Attenuates Glioma Growth. Glia 67, 2424–2439. 10.1002/glia.23696 31400163 PMC7008520

[B125] Wang W. Caldwell M. C. Lin S. Furneaux H. Gorospe M. (2000). HuR Regulates Cyclin A and Cyclin B1 mRNA Stability during Cell Proliferation. Embo j 19, 2340–2350. 10.1093/emboj/19.10.2340 10811625 PMC384372

[B126] Wang Y. Gao R. Li J. Tang S. Li S. Tong Q. (2021). Downregulation of Hsa_circ_0074854 Suppresses the Migration and Invasion in Hepatocellular Carcinoma via Interacting with HuR and via Suppressing Exosomes-Mediated Macrophage M2 Polarization. Ijn 16, 2803–2818. 10.2147/ijn.S284560 33880025 PMC8052130

[B127] Wang Y. Guo Y. Tang C. Han X. Xu M. Sun J. (2019). Developmental Cytoplasmic-To-Nuclear Translocation of RNA-Binding Protein HuR Is Required for Adult Neurogenesis. Cell Rep. 29, 3101–3117. e3107. 10.1016/j.celrep.2019.10.127 31801076

[B128] Wen H. Chen Z. Cui Y. Xu Y. (2021). LncRNA NONHSAT009968 Inhibits the Osteogenic Differentiation of hBMMSCs in SA-Induced Inflammation via Wnt3a. Biochem. Biophysical Res. Commun. 577, 24–31. 10.1016/j.bbrc.2021.08.086 34492499

[B129] Woo H.-H. Zhou Y. Yi X. David C. L. Zheng W. Gilmore-Hebert M. (2009). Regulation of Non-AU-rich Element Containing C-Fms Proto-Oncogene Expression by HuR in Breast Cancer. Oncogene 28, 1176–1186. 10.1038/onc.2008.469 19151756

[B130] Wu X. Gardashova G. Lan L. Han S. Zhong C. Marquez R. T. (2020). Targeting the Interaction between RNA-Binding Protein HuR and FOXQ1 Suppresses Breast Cancer Invasion and Metastasis. Commun. Biol. 3, 193. 10.1038/s42003-020-0933-1 32332873 PMC7181695

[B131] Wu X. Lan L. Wilson D. M. Marquez R. T. Tsao W.-c. Gao P. (2015). Identification and Validation of Novel Small Molecule Disruptors of HuR-mRNA Interaction. ACS Chem. Biol. 10, 1476–1484. 10.1021/cb500851u 25750985 PMC4631057

[B132] Xie M. Ma T. Xue J. Ma H. Sun M. Zhang Z. (2019). The Long Intergenic Non-protein Coding RNA 707 Promotes Proliferation and Metastasis of Gastric Cancer by Interacting with mRNA Stabilizing Protein HuR. Cancer Lett. 443, 67–79. 10.1016/j.canlet.2018.11.032 30502359

[B133] Yang F. Hu A. Li D. Wang J. Guo Y. Liu Y. (2019). Circ-HuR Suppresses HuR Expression and Gastric Cancer Progression by Inhibiting CNBP Transactivation. Mol. Cancer 18, 158. 10.1186/s12943-019-1094-z 31718709 PMC6852727

[B134] Yano M. Hayakawa‐Yano Y. Okano H. (2016). RNA Regulation Went Wrong in Neurodevelopmental Disorders: The Example of Msi/Elavl RNA Binding Proteins. Int. J. Dev. Neurosci. 55, 124–130. 10.1016/j.ijdevneu.2016.01.002 26796049

[B135] Young L. E. Sanduja S. Bemis–Standoli K. Pena E. A. Price R. L. Dixon D. A. (2009). The mRNA Binding Proteins HuR and Tristetraprolin Regulate Cyclooxygenase 2 Expression during colon Carcinogenesis. Gastroenterology 136, 1669–1679. 10.1053/j.gastro.2009.01.010 19208339 PMC3742387

[B136] Zang Y. Li J. Wan B. Tai Y. (2020). circRNA circ‐CCND1 Promotes the Proliferation of Laryngeal Squamous Cell Carcinoma through Elevating CCND1 Expression via Interacting with HuR and miR‐646. J. Cell Mol Med 24, 2423–2433. 10.1111/jcmm.14925 31951319 PMC7028846

[B137] Zaragozá C. Álvarez-Mon M. Á. Zaragozá F. Villaescusa L. (2022). Flavonoids: Antiplatelet Effect as Inhibitors of COX-1. Molecules 27, 1146. 10.3390/molecules27031146 35164411 PMC8839657

[B138] Zhang C. Han X. Yang L. Fu J. Sun C. Huang S. (2020). Circular RNA circPPM1F Modulates M1 Macrophage Activation and Pancreatic Islet Inflammation in Type 1 Diabetes Mellitus. Theranostics 10, 10908–10924. 10.7150/thno.48264 33042261 PMC7532688

[B139] Zhang L. F. Lou J. T. Lu M. H. Gao C. Zhao S. Li B. (2015). Suppression of miR‐199a Maturation by HuR Is Crucial for Hypoxia‐induced Glycolytic Switch in Hepatocellular Carcinoma. Embo j 34, 2671–2685. 10.15252/embj.201591803 26346275 PMC4641532

[B140] Zhang X. Lee M. D. Buckley C. Wilson C. McCarron J. G. (2022). Mitochondria Regulate TRPV4‐mediated Release of ATP. Br. J Pharmacol. 179, 1017–1032. 10.1111/bph.15687 34605007

[B141] Zhao Q. Li C. Yu M. Sun Y. Wang J. Ma L. (2020). HuR Stabilizes HTT mRNA via Interacting with its Exon 11 in a Mutant HTT-dependent Manner. RNA Biol. 17, 500–516. 10.1080/15476286.2020.1712894 31928144 PMC7237150

[B142] Zhao W. S. Yan W. P. Chen D. B. Dai L. Yang Y. B. Kang X. Z. (2019). Genome-scale CRISPR Activation Screening Identifies a Role of ELAVL2-Cdkn1a axis in Paclitaxel Resistance in Esophageal Squamous Cell Carcinoma. Am. J. Cancer Res. 9, 1183–1200. https://www.ncbi.nlm.nih.gov/labs/pmc/articles/PMC6610048/pdf/ajcr0009-1183.pdf. 31285951 PMC6610048

[B143] Zhao Y.-F. He X.-X. Song Z.-F. Guo Y. Zhang Y.-N. Yu H.-L. (2020). Human Antigen R-Regulated mRNA Metabolism Promotes the Cell Motility of Migrating Neurons. Development 147. 10.1242/dev.183509 PMC7097226 32098764

[B144] Zhou H. Telonis A. G. Jing Y. Xia N. L. Biederman L. Jimbo M. (2016). GPRC5A Is a Potential Oncogene in Pancreatic Ductal Adenocarcinoma Cells that Is Upregulated by Gemcitabine with Help from HuR. Cell Death Dis 7–e2294. 10.1038/cddis.2016.169 PMC4973341 27415424

[B145] Zhu Y. Yang L. Xu J. Yang X. Luan P. Cui Q. (2020). Discovery of the Anti-angiogenesis Effect of Eltrombopag in Breast Cancer through Targeting of HuR Protein. Acta Pharmaceutica Sinica B 10, 1414–1425. 10.1016/j.apsb.2020.02.007 32963940 PMC7488360

