# Supplementary material for: Roles of Embryonic Lethal Abnormal Vision-Like RNA Binding Proteins in Cancer and Beyond
Source: Front Cell Dev Biol. 2022 Apr 6;10:847761. doi: 10.3389/fcell.2022.847761 (PMC9019298; doi:10.3389/fcell.2022.847761)
Supplement: Supplementary file 1 [file Table1.docx]

**Supplementary Table 1 MRNA interacting with ELAVL1**

| Member of protein family | Target | Positive/Negative regulation (P/N) | Disease model/cell type | PMID |
| --- | --- | --- | --- | --- |
| ELAVL1 | VEGF | P | gastric carcinoma | 32600329 |
| ELAVL1 | VEGF | P | Tumor endothelial cells | 21285980 |
| ELAVL1 | VEGF | P | Malignant brain tumor | 11280780 |
| ELAVL1 | VEGF | P | HEK 293 cells | 9497373 |
| ELAVL1 | VEGF | P | Cervical cancer HeLa cells | 26325091 |
| ELAVL1 | VEGF | P | HEK 293 cells | 21723170 |
| ELAVL1 | VEGF | P | Pancreatic ductal adenocarcinoma | 20739850 |
| ELAVL1 | VEGF | Unclear | Rat Nucleus pulposus (NP) cells | 30092282 |
| ELAVL1 | DNMT3 | P | Breast cancer | 31636039 |
| ELAVL1 | DNMT3 | P | Colorectal cancer | 19270063 |
| ELAVL1 | YAP | P | malignant peripheral nerve sheath tumor | 32315290 |
| ELAVL1 | YAP | P | Osteosarcoma | 30182452 |
| ELAVL1 | CCND1 | P | malignant peripheral nerve sheath tumor | 32315290 |
| ELAVL1 | CCND1 |  | Cervical cancer HeLa cells | 15257295 |
| ELAVL1 | CCND2 | P | malignant peripheral nerve sheath tumor | 32315290 |
| ELAVL1 | CCND2 | P | osteosarcoma | 30102375 |
| ELAVL1 | CDK2 | P | malignant peripheral nerve sheath tumor | 32315290 |
| ELAVL1 | CDK6 | P | malignant peripheral nerve sheath tumor | 32315290 |
| ELAVL1 | p27 | P | malignant peripheral nerve sheath tumor | 32315290 |
| ELAVL1 | p27 | P | glioblastoma cell | 26874904 |
| ELAVL1 | p27 | P | Bone marrow differentiated macrophages (BMDMs) | 33262333 |
| ELAVL1 | E2F1 | P | malignant peripheral nerve sheath tumor | 32315290 |
| ELAVL1 | E2F2 | P | malignant peripheral nerve sheath tumor | 32315290 |
| ELAVL1 | E2F3 | P | malignant peripheral nerve sheath tumor | 32315290 |
| ELAVL1 | CTNNB1 | P | malignant peripheral nerve sheath tumor | 32315290 |
| ELAVL1 | CTNNB1 | P | mammary cancer | 24954509 |
| ELAVL1 | BRD2, BRD3, BRD4 | P | malignant peripheral nerve sheath tumor | 32315290 |
| ELAVL1 | MMP-9 | P | mesangial cells | 14523003 |
| ELAVL1 | MMP-9 | P | pterygium fibroblast | 31691974 |
| ELAVL1 | MMP-9 | P | Primary rat hippocampal neuronal | 29686606 |
| ELAVL1 | C/EBP-α , PPAR-γ | P | Cancer-related cachexia (CAC) | 30807648 |
| ELAVL1 | PGC-1α | P | C2C12 myoblasts | 31519904 |
| ELAVL1 | CDC6 | P | Colorectal Cancer | 31064870 |
| ELAVL1 | MSH3, CCR6 | P | osteosarcoma | 30102375 |
| ELAVL1 | IDH1 | P | Pancreatic Cancer Cells | 28652247 |
| ELAVL1 | PARG | P | Pancreatic ductal adenocarcinoma | 28687616 |
| ELAVL1 | caspase-2 | P | colorectal cancer cells | 28219770 |
| ELAVL1 | GPRC5A | P | pancreatic cancer | 27415424 |
| ELAVL1 | WNT5A | P | melanoma cell | 26970271 |
| ELAVL1 | PIM1 | P | pancreatic cancer | 26387536 |
| ELAVL1 | p53 | P | MCF7 | 27592685 |
| ELAVL1 | TJP1,SOX4,SOX9,MDM2,KRAS,p53,BAX | Unclear | HCT116 | 21317874 |
| ELAVL1 | COX-2 | P | Tumor endothelial cells | 21285980 |
| ELAVL1 | ATF2,  CTGF, RAB31 | Unclear | Breast cancer | 21480233 |
| ELAVL1 | MAT2A | P | Hepatocellular Carcinoma | 20102719 |
| ELAVL1 | TUBB,  TUBB3 | P | ovarian cancer | 20587520 |
| ELAVL1 | COX-2 | P | colon carcinogenesis | 19208339 |
| ELAVL1 | COX-2 | P | Prostate cancer | 18468781 |
| ELAVL1 | COX-2 | P | mesothelioma | 18831511 |
| ELAVL1 | COX-2 | P | Aortic smooth muscle | 25653183 |
| ELAVL1 | c-fms | P | breast cancer | 19151756 |
| ELAVL1 | c-Myc | N | Cervical cancer HeLa cells | 19574298 |
| ELAVL1 | c-Myc | P | Mouse embryo | 11103939 |
| ELAVL1 | dCK | P | pancreatic cancer | 19487279 |
| ELAVL1 | ELAVL1 | P | Cervical cancer HeLa cells | 19359363 |
| ELAVL1 | TSP1 | P | MCF7 | 18641687 |
| ELAVL1 | Wnt-5a | N | breast cancer | 16914445 |
| ELAVL1 | cyclin E1 | P | MCF7 | 16912169 |
| ELAVL1 | IL-8 | P | breast cancer | 15514971 |
| ELAVL1 | ProTalpha | P | Cervical cancer HeLa cells | 15861128 |
| ELAVL1 | GM-CSF | P | HT1080 | 14976220 |
| ELAVL1 | p53 | P | Colorectal cancer | 12821781 |
| ELAVL1 | beta-catenin | P | Colorectal cancer | 14562043 |
| ELAVL1 | cyclin A | P | Colorectal cancer | 10811625 |
| ELAVL1 | cyclin B1 | P | Colorectal cancer | 10811625 |
| ELAVL1 | cyclin B1 | P | Cervical epithelial cells | 16458113 |
| ELAVL1 | TNF-α | P | Cervical cancer HeLa cells | 9925643 |
| ELAVL1 | p27kip1 | P | Cervical cancer HeLa cells | 10908325 |
| ELAVL1 | IGF-IR | N | OVCAR3 cells | 15914670 |
| ELAVL1 | SIRT1 | P | Cervical cancer HeLa cells | 17317627 |
| ELAVL1 | Cx43 | P | oval cell-like rat liver epithelial cells | 19676129 |
| ELAVL1 | beta-catenin | P | oval cell-like rat liver epithelial cells | 19676129 |
| ELAVL1 | XIAP | P | HEK 293 cells | 21102524 |
| ELAVL1 | HNRNPK | N | Cervical cancer HeLa cells | 21723171 |
| ELAVL1 | AChR β-subunit | P | Mouse tibial anterior muscle cells | 26245959 |
| ELAVL1 | COQ7 | P | Cervical cancer HeLa cells | 26690054 |
| ELAVL1 | MR | P | HEK 293 cells | 28744670 |
| ELAVL1 | TIN2 | N | Cervical cancer HeLa cells | 29584879 |
| ELAVL1 | Pabpn1 | N | C2C12 myoblasts | 29939290 |
| ELAVL1 | VHL | P | Cervical cancer HeLa cells | 24106086 |
| ELAVL1 | Cxcl2 | P | HEK 293 cells | 28272405 |
| ELAVL1 | sGCα 1, sGCβ 1 | P | Rat aorta | 15883232 |
| ELAVL1 | TLR4 | P | Human arterial smooth muscle cells | 16990552 |
| ELAVL1 | KLF2, eNOS, | N | Atherosclerosis | 20351266 |
| ELAVL1 | BMP-4 | P | Atherosclerosis VEGFA | 20351266 |
| ELAVL1 | CTSS | P | Human umbilical vein endothelial cells | 27595325 |
| ELAVL1 | CUGBP1 | N | Myocardial infarction | 28350193 |
| ELAVL1 | SCN5A | P | Human fetal cardiomyocyte cell line RL14 | 29454929 |
| ELAVL1 | TGF-β | P | pathological cardiac hypertrophy | 30668549 |
| ELAVL1 | CD62E, cathepsin S | P | Mouse endothelial cells | 29970364 |
| ELAVL1 | SGLT1 | P | ischaemia/reperfusion injury | 30715251 |
| ELAVL1 | p21 | P | H1299 (non-small cell lung cancer) | 15371446 |
| ELAVL1 | PLB | P | Rat cardiomyocyte H9C2 cell | 31373621 |
| ELAVL1 | β1-AR | N | Rat cardiomyocyte H9C2 cell | 31373621 |
| ELAVL1 | RGS2, RGS4, RGS5 | P | Mouse aortic smooth muscle cells | 32075416 |
| ELAVL1 | SMN | P | Spinal muscle atrophy (SMA) | 19648294 |
| ELAVL1 | CCNA2 | P | HEK 293 cells | 21723170 |
| ELAVL1 | cyclin D1, MCP-1 | P | Hepatic stellate cell | 22576182 |
| ELAVL1 | IL-20, CXCL-10, DEFB4A, and SLC6A14 | P | Keratinocytes | 26176762 |
| ELAVL1 | NOD2 | P | Rat glomerular mesangial cells (RMCs) | 25528059 |
| ELAVL1 | Bcl2, NQO1, Ppargc1a | P | HT-22 cells, mouse hippocampal neuron cells | 25301069 |
| ELAVL1 | Cirbp, Gja1 | N | HT-22 cells, mouse hippocampal neuron cells | 25301069 |
| ELAVL1 | FMR1 | P | Lymphoblast | 26554012 |
| ELAVL1 | Cnpy3 | P | Paneth cell | 31103627 |
| ELAVL1 | Insig1 | P | subcutaneous white preadipocytes | 31924774 |
| ELAVL1 | Pfn1 | P | Mouse neuron cell | 32098764 |
| ELAVL1 | TNF-α | P | Murine macrophage cell line B10R | 11160917 |
| ELAVL1 | IL-6, CXCL, Trem, MMP12,MMP13, IL-1β | P | microglia | 28300326 |
| ELAVL1 | p62 | P | ARPE-19 Cells | 29576851 |
| ELAVL1 | TERC | P | HEK 293 cells | 29880812 |
| ELAVL1 | Beclin1 | P | Hepatic stellate cells | 30081711 |
| ELAVL1 | ATGL | P | 3T3-L1 cells | 31147543 |
| ELAVL1 | STAT3 | P | C2C12 cells | 31405989 |
| ELAVL1 | HIF-1α, COL1A1 | Unclear | Nucleus pulposus cells | 30092282 |
| ELAVL1 | HO-1 | P | Hepatocyte | 31879990 |
| ELAVL1 | IL-17 | P | mouse podocytes | 32487989 |
| ELAVL1 | claudin-1 | P | Colorectal cancer | 23880304 |
| ELAVL1 | PAI-1 | P | Glomerulosclerosis | 32478392 |
| ELAVL1 | PPM1F | P | M1 macrophage | 33042261 |
| ELAVL1 | APOB, UQCRB, NDUFB6 | P | Hepatocyte | 32546794 |
| ELAVL1 | HTT | P | Human fibroblast | 31928144 |
| ELAVL1 | Atg7 | P | Nucleus pulposus | 33372336 |
| ELAVL1 | c-fos | P | NIH 3T3 cells | 9628881 |
| ELAVL1 | Plk2 | P | HEK 293 cells | 29678949 |
| ELAVL2 | c-myc | Unclear | COS cells | 8497264 |
| ELAVL2 | c-myc | P | 3T3-L1 cells | 9763509 |
| ELAVL2 | GLUT1 | P | 3T3-L1 cells | 9763509 |
| ELAVL2 | MYCN | P | Neuroblastoma | 16912187 |
| ELAVL2 | c-myc,  c-fos, GM-CSF | N | Human medulloblastoma cell lines D283 and D341 | 7972035 |
| ELAVL2 | N-myc | Unclear | Human neuroblastoma | 8969226 |
| ELAVL2 | NF-M | P | hNT2 cells | 10049360 |
| ELAVL2 | l-myc,, b-myc, max, and cyclins A2, B1, C, D1 and D2 | Unclear | Mouse P19 cells | 11121017 |
| ELAVL2 | Foxg1 | P | P19 cells | 21368052 |
| ELAVL2 | Gap43 | P | Hippocampal CA3 pyramidal neurons | 21695151 |
| ELAVL2 | MMP-9 | P | Primary rat hippocampal neuronal | 29686606 |
| ELAVL2 | CDKN1A | P | ESCC cell line | 31285951 |
| ELAVL2 | SNAI2 | P | HTM cells | 33522089 |
| ELAVL3 | c-myc, VEGF | Unclear | Patient serum | 10710437 |
| ELAVL3 | GAP-43 | P | Cells-IIC9 cells | 11573004 |
| ELAVL3 | TNF-α,  c-myc | Unclear | neuroblastoma cell line IMR-32 | 10100634 |
| ELAVL4 | p21(waf1) | Unclear |  | 9685407 |
| ELAVL4 | c-myc, VEGF | Unclear | Patient serum | 10710437 |
| ELAVL4 | N-myc | P | Neuroblastoma | 16912187 |
| ELAVL4 | GAP-43 | P | Neurons | 29061699 |
| ELAVL4 | p27 | P | Pancreatic neuroendocrine tumors | 30014466 |
| ELAVL4 | tau | P | PC12 cells | 10436048 |
| ELAVL4 | AChE | P | Superior cervical ganglion | 17234598 |
| ELAVL4 | β-actin | P | L2-6 DRGs | 26152301 |
| ELAVL4 | SATB1 | P | neural stem/progenitor cells | 26305964 |
| ELAVL4 | cpg15 | P | Neurons | 21652774 |
| ELAVL4 | NRN1 (CPG15) | P | Motor neuron | 28988989 |
| ELAVL4 | Eef1a1, Pabpc1, Eif4a1, Eif4a2 | P | Motor neuron | 30029004 |
| ELAVL4 | Ins2 | N | mouse insulinoma βTC6 cells | 33338402 |
